# Androgen Deprivation Therapy for Prostate Cancer Is Associated with Cardiovascular Morbidity and Mortality: A Meta-Analysis of Population-Based Observational Studies

**DOI:** 10.1371/journal.pone.0107516

**Published:** 2014-09-29

**Authors:** Jinsheng Zhao, Shimiao Zhu, Libin Sun, Fanzheng Meng, Lin Zhao, Yusheng Zhao, Hao Tian, Ping Li, Yuanjie Niu

**Affiliations:** 1 Tianjin Medical University, Tianjin, China; 2 Department of Neurology, Tianjin Nankai Hospital, Tianjin, China; 3 Department of Urology, Tianjin Institute of Urology, Second Hospital of Tianjin Medical Unversity, Tianjin, China; 4 First teaching hospital of Tianjin University of TCM, Tianjin, China; 5 Tianjin Third Central Hospital, Tianjin, China; University of Kentucky College of Medicine, United States of America

## Abstract

**Background:**

There is no consensus regarding whether androgen deprivation therapy (ADT) is associated with cardiovascular disease (CVD) and cardiovascular mortality (CVM). The objective of this study was to determine the role of ADT for prostate cancer (PCa) in development of cardiovascular events (CVD and CVM).

**Methods and Findings:**

We performed a meta-analysis from population-based observational studies comparing ADT vs control aimed at treating PCa in patients with PCa, reporting either CVD or CVM as outcome. Publications were searched using Medline, Embase, Cochrane Library Central Register of observational studies database up to May 31th 2014, and supplementary searches in publications from potentially relevant journals. 6 studies were identified with a total of 129,802 ADT users and 165,605 controls investigating the relationship between ADT and CVD. The incidence of CVD was 10% higher in ADT groups, although no significant association was observed (HR = 1.10, 95%CIs: 1.00–1.21; P = 0.06). For different types of ADT, CVD was related with gonadotropin-releasing hormone (GnRH) (HR = 1.19, 95%CIs: 1.04–1.36; P<0.001) and GnRH plus oral antiandrogen (AA) (HR = 1.46, 95%CIs: 1.03–2.08; P = 0.04), but not with AA alone or orchiectomy. For CVM, 119,625 ADT users and 150,974 controls from 6 eligible studies were included, pooled results suggested that ADT was associated with CVM (HR = 1.17, 95%CIs: 1.04–1.32; P = 0.01). Significantly increased CVM was also detected in GnRH and GnRH plus AA groups. When patients received other treatments (e.g. prostatectomy and radiotherapy) were ruled out of consideration, more increased CVD (HR = 1.19, 95%CIs: 1.08–1.30; P<0.001) and CVM (HR = 1.30, 95%CIs: 1.13–1.50; P<0.001) were found in men treated with ADT monotherapy.

**Conclusions:**

ADT is associated with both CVD and CVM. Particularly, GnRH alone and GnRH plus AA can significantly increase the incidence of cardiovascular events in patients with PCa.

## Introduction

Prostate cancer (PCa) remains one of the most common type of solid malignancy and the second-leading cause of all cancer death among US men [1]. Pivotal studies [2], [3] demonstrated that the development and growth of PCa cells are dependent on androgens. Since then, androgen deprivation therapy (ADT) has been increasingly used as the treatment of PCa.

Despite its well-recognized efficacy, ADT is not an innocuous therapy:It may increase the risk of cardiovascular disease (CVD) and cardiovascular mortality (CVM). One nation-wide cohort study [4] demonstrated that ADT is significantly related to a greater incidence of CVD. Another study [5] reported an increased risk of CVM over a median follow-up of 2.6 years in men treated with gonadotropin-releasing hormone agonist (HR, 1.28, 95% CI, 1.05–1.57).

Conflicting results were reported in some other papers. A population-based cohort study found that ADT was not associated with acute myocardial infarction or sudden cardiac death [6]. Particularly, a meta-analysis [7] of 8 randomized controlled trials (RCTs) about cardiac death related to ADT in patients with localized PCa drew a non-significant conclusion that ADT tended to be associated with an increased risk of CVM (RR, 0.93; 95% CI, 0.79–1.10; *P = 0.41*). But the validity of the results in this meta-analysis was suspected [8] because of the following bias: (1) ADT was administered for a time interval that was less than the duration of data collection in 6 of the 8 studies, thereby introducing timing bias; (2) 4 of the studies [9]–[12] in the meta-analysis have contamination bias, many patients in the control group eventually received ADT; (3) 6 studies have confounding bias, more than one type of ADT was used in treatment groups, but subgroup analysis of different types of ADT was not performed. Because of all these biases listed above, it is inappropriate to use RCTs to test whether or not there is an association between ADT and CVM. Additionally, as a rare adverse effect (12.9/1000 person-years [13]), cardiovascular events are not the main endpoint RCTs always focus on. For the purpose of investigating rare adverse drug reactions, it is more credible to perform a meta-analysis of large-scale observational studies with long duration of follow-up, high quality of design and implementation [14].

Based on the controversy of this clinical issue and the limitation in meta-analysis of RCTs for adverse-effect research, a comprehensive meta-analysis of population-based observational studies was performed by our research group to explore whether ADT as well as different types of this treatment is associated with CVD or CVM in patients with PCa.

## Methods

### Search Strategy and Study Selection

A systematic search of MEDLINE, EMBASE and the Cochrane Library database was performed through May 31^th^, 2014, using all possible combinations of the following keywords: *prostate cancer* or *prostate tumor* or *prostate carcinoma*, *androgen* and *deprivation* or *androgen suppression* or *endocrine treatment*; and *cardiovascular* or *myocardial infarction* or *coronary heart disease* or *cardiac death* or *heart disease* (Methods S1 in File S1). To perform the extensive search, there was no language, publication year, or other limit used. References of included studies and narrative reviews were searched for potential studies.

Included studies were restricted to observational studies that should meet all of the following inclusion criteria: (1) The type of studies should be population-based cohort or nested case-control without subjects-selection bias; (2) Patients diagnosed with PCa; (3) The intervention groups used ADT only or ADT plus other treatments; (4) The patients in control groups never received ADT; (5) Study must either report risk estimates with 95% Confidence intervals (CIs), or report sufficient data to estimate these; (6) Included studies had to provide comparative data. If more than one paper were identified from the same database, the most measurable (complete or recent) report of these articles was chosen for analysis.

### Data Extraction and Quality Assessment

All the data from eligible publications were carefully extracted independently by two authors (Zhao & Zhu), and all disagreements were resolved by the third reviewer (Niu) until consensus was achieved on all items. We inspected analyses of the individual participant data for consistency with each published reports to ensure that the data were incorporated correctly into this meta-analysis. For each study included, the following information was considered: first author's name, year and research institution of the study, the number of case and control patients, median age of patients, duration of follow-up, inclusion criteria, treatment in both groups, types and duration of ADT, definition of cardiovascular events, hazard ratios (HRs) or risk ratios (RRs) and corresponding 95% CIs of estimates in each comparisons, or the information required to calculate these. The definition of cardiovascular event was consistent with description in each eligible study. Any type of ADT was included in our meta-analysis involving gonadotropin-releasing hormone (GnRH) agonists, oral antiandrogens (AA), orchiectomy, and combined ADT (two or more types above combined).

Eligible studies were assessed by the Newcastle-Ottawa quality assessment scale (NOS) [15]. The observational studies were considered to be of high-quality if it achieved more than six stars. Assessments were addressed respectively by two reviewers (Zhao & Zhu) and discrepancies were discussed until agreement was met. In addition, level of evidence (LOE) of all included studies were assessed according to the classifications by Phillips et al. [16].

### Statistical Analysis

The HRs were used to compare all dichotomous variables. As described in detail previously [17], we used different methods to estimate the HRs according to the data provided in the publications. When two or more types of ADT from one study were respectively compared with the same control group (e.g. GnRH vs Control and AA vs Control), we used random effects meta-analysis to combine these different types of ADT groups as necessary.

Cochrane's Q statistic [18] was used to assess the statistical heterogeneity between included studies. Additionally, inconsistency was quantified by *I* ^2^ statistic (100%× [(Q-df)/Q]), and a higher value indicates a greater degree of heterogeneity [19]. The assumption of homogeneity was considered invalid for *P*<0.05. With the Der-Simonian and Laird method, random-effects model was used no matter whether heterogeneity was observed or not.

Begg adjusted rank correlation test [20] and Egger linear regression test [21] were used to evaluate publication bias. Statistical analyses were conducted with Review Manager (version 5.2; The Cochrane Collaboration, Oxford) and STATA (version 11.0; College Station, Texas). Two-tailed *P*<0.05 was deemed to be statistically significant.

### Subgroups Analyses

To minimize the influence of prior treatments (e.g. radiotherapy, prostatectomy, etc.), analyses of ADT monotherapy vs watchful waiting or active surveillance (WW/AS) for cardiovascular events were particularly performed. ADT monotherapy was defined as treatment in intervention group was only ADT without any other previous therapies. Additionally, subgroup analyses for different types of ADT (e.g. GnRH, AA, GnRH +AA and Orchiectomy) vs non-ADT were performed to minimize existing heterogeneity.

## Results

### Literature Search and Characteristics of the Included Studies

**Figure 1:**
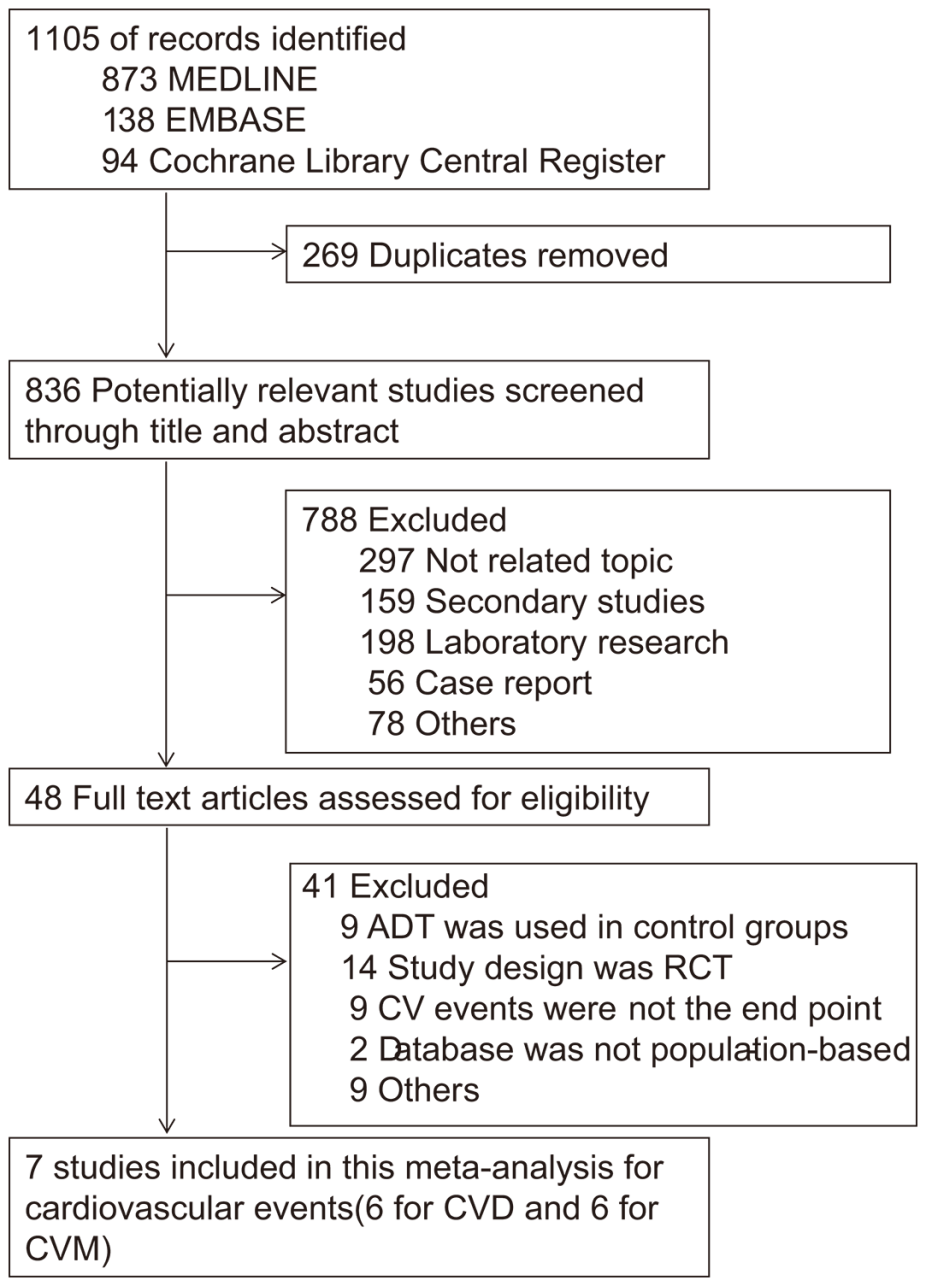
Flow Diagram of Search Strategy and Study Selection.

After removing 269 duplicates, we identified 836 unique articles from Medline, Embase and Cochrane Library. We then excluded 788 studies by carefully reading the title and abstract. Based on the full text screening from the remaining 48 articles, we further removed 9 studies which did not mention cardiovascular events as endpoint and 9 with ADT also used in control group. Additionally, 14 RCTs were excluded from our study. For the remaining 16 studies, one study [22] was excluded for using the same database as Punnen et al.'s [23] with less complete data. One [24] of these four studies [4], [13], [24], [25] using the same database were included because of more complete and recent data. Meanwhile, among another three studies [26]–[28] coming from the same database, Hemelrijck et al.'s [28] was included at last for the complete data. Two [29], [30] were excluded because the database were not population-based. The last one [31] was excluded because of selection bias (only patients underwent external beam radiation therapy were included). Finally, 7 studies were included in this meta-analysis (Figure 1). All papers used in our analysis were published in English. List of excluded full-text articles with reasons are shown in Table S1 in File S1. Among these eligible studies, one [32] was nested case-control study, all the others were cohort studies. HRs and 95% CIs reported in two studies [6], [23] were directly extracted.

Search of the references listed in reviews did not yield any further studies for evaluation. Table 1 and Table 2 summarized the characteristics of included studies for CVD and CVM. The results of quality assessment according to NOS were shown in Table S2 in File S1. All eligible studies were of high quality based on NOS with scores ranged from six to nine stars. LOE of all studies were 2a.

**Table 1:** Characteristics of Studies Investigating CVD Related to ADT.

First author, year	Design, LOE	Database source (Duration)	Definition of CVD (ICD codes)	Types of ADT	Treatment in control group	No. of ADT	No. of Control	Age y^a^(SD)/No. of patients	Follow-up (y)^a^	Adjusted HRs(95%CI)	
Jespersen et al., [33] 2013	Cohort, 2a	Danish Cancer Registry (2002–2010)	AMI (ICD-8 codes 410.09/410.99 and IDC-10 codes DI21.x)	GnRH/AA Orchiectomy	non-ADT	9,204 2,060	20,307	71	3.3 (1.8 to 5.2)	1.31 (1.16,1.49)^c^ 0.90 (0.83,1.29)^c^	1.19 (0.94, 1.50)^d^
Hemelrijck et al., [28] 2010	Cohort, 2a	NPCR of Sweden (1997–2007)	Ischemic Heart Disease (ICD-10: I20 through I25)	GnRH agonist AA GnRH + AA Orchiectomy Other types	RP WW/AS	9,066 3,391 11,646 5,340 1,199	26,432 19,526	≤65: 19153 66 to 74: 27737 ≥75: 13110	4.1	1.28 (1.20,1.37)^b^ 0.91 (0.82,1.02)^b^ 1.18 (1.11,1.26)^b^ 1.37 (1.26,1.48)^b/^	1.18 (1.03, 1.35)^d^
Merino et al., [32] 2011	Nested Case-Control, 2a	GPRD (1999–2005)	Hospitalization from AMI (NA)	GnRH agonist AA GnRH + AA Orchiectomy	WW/AS	591 381 431 10	335	51 to 69: 324 70 to 84: 850	NA	1.26 (0.91,1.73)^b^ 0.86 (0.45,1.63)^b^ 1.17 (0.83,1.65)^b^ 0.81 (0.13,5.24)^b^	1.26 (0.78, 2.20)^c^
Alibhai et al., [6] 2009	Cohort, 2a	ICES (1995–2005)	AMI (ICD-9-CM 410.0–410.9)	ADT	non-ADT	19079	19,079	75±6.3	6.47	0.92(0.84,1.00)^c^	
Keating et al., [5] 2010	Cohort, 2a	Veterans Healthcare Administration (2001–2004)	CHD (ICD-9 codes 411–414.9 except 414.1X)	GnRH agonist AA GnRH + AA Orchiectomy	WW/AS	13,065 1,230 1,829 268	23,823	66.9±8.6	2.6	1.21 (1.06,1.39)^c^ 1.01 (0.85,1.20)^c^ 1.29 (1.00,1.66)^c^ 1.48 (1.00,2.20)^c^	1.18 (1.03, 1.35)^d^
Gandaglia et al., [24] 2014	Cohort, 2a	SEER (1995–2009)	CVD (ICD-9)	GnRH agonist Orchiectomy	non-ADT	57,939 2,055	82,535	73.6 (69 to 77)	6.28	1.11 (1.07–1.15)^c^ 1.02 (0.88–1.18)^c^	1.10 (1.04, 1.16)^d^

Abbreviations: LOE =  level of evidence; CVD =  cardiovascular disease; ADT =  androgen deprivation therapy;

GnRH =  gonadotropin-releasing hormone (leuteinizing hormone releasing hormone, LHRH); AA =  oral antiandrogens; RP =  Radical prostatectomy/Curative Treatment; WW/AS =  watchful waiting (WW)/active surveillance (AS); AMI =  Acute Myocardial Infarction; CHD =  Coronary Heart Disease; HRs =  Hazard Ratios; RRs =  Risk Ratios; NA =  not applicable; NPCR =  National Prostate Cancer Register; SEER =  Surveillance, Epidemiology and End Results Medicare data; GPRD  =  UK General Practice Research Database; ICES  =  Institute for Clinical Evaluative Sciences.

a mean or median.

b compared with WW/AS.

c The HR/RR was directly given in the publication.

d Combined estimates from all types of ADT with random effects meta-analysis.

**Table 2:** Characteristics of Studies Investigating CVM Related to ADT.

First author, year	Design, LOE	Database source (Duration)	Definition of CVD (ICD codes)	Types of ADT	Treatment in control group	No. of ADT	No. of Control	Age y^a^(SD)/No. of patients	Follow-up (y)^a^	Adjusted HRs(95%CI)	
Punnen et al., [23] 2011	Cohort, 2a	CaPSURE (1995–2007)	CVM: AMI, cardiac ischemia, sudden cardiac arrest or death, coronary artery disease, or alignant arrhythmia(NA)	ADT only ADT + RP/RT	RP/RT WW/AS	1,087 485	5,170 506	≤65: 3390 >65: 3858	4.38/4.27 4.75/3.97	1.12(0.73, 1.67)^c^	
Alibhai et al., [6] 2009	Cohort, 2a	ICES (1995–2005)	CHD (ICD-9 codes 411–414.9 except 414.1X)	ADT	non-ADT	19,079	19,079	75±6.3	6.47	0.96(0.83,1.10)^c^	
Hemelrijck et al., [28] 2010	Cohort, 2a	NPCR of Sweden (1997–2007)	Ischemic Heart Disease (ICD-10: I20 through I25)	GnRH agonist Orchiectomy AA GnRH +AA	WW/AS RP	9066 5340 3391 11646	19526 26432	≤65: 19153 66 to 74: 27737 ≥75: 13110	4.1	1.57(1.44,1.72)^b^ 2.07(1.88,2.28)^b^ 0.75(0.63,0.89)^b^ 1.45(1.33,1.78)^b^	1.38 (1.02,1.87)^d^
Gandaglia et al., [24] 2014	Cohort, 2a	SEER (1995–2009)	CVD (ICD-9)	GnRH agonist Orchiectomy	non-ADT	57,939 2,055	82,535	73.6 (69 to 77)	6.28	1.18(1.12–1.24)^c^ 1.15(0.97–1.38)^c^	1.18 (1.12,1.24)^d^
Keating et al., [5] 2010	Cohort, 2a	Veterans Healthcare Administration (2001–2004)	CHD (ICD-9 codes 411–414.9 except 414.1X)	GnRH agonist Orchiectomy AA CAB	WW/AS	13065 268 1230 1829	23823	66.9±8.6	2.6	1.28(1.05,1.57)^c^ 1.70(0.86,3.34)^c^ 1.47(0.69,3.14)^c^ 1.05(0.60,1.87)^c^	1.29 (1.08,1.55)^d^
Merino et al., [32] 2011	Nested Case-Control, 2a	GPRD (1999–2005)	death due to Coronary Heart Disease (NA)	GnRH agonist Orchiectomy AA GnRH +AA	WW/AS	591 10 481 431	335	51 to 69: 324 70 to 84: 850	NA	2.15(0.81,5.72) 2.10(0.42,10.53) 1.04(0.99,1.10) 2.02(0.73,5.61)	1.65 (0.87,3.13)^d^

Abbreviations: LOE =  level of evidence; CVM =  cardiovascular mortality; ADT =  androgen deprivation therapy; CAB =  combined androgen blockade; GnRH =  gonadotropin-releasing hormone (leuteinizing hormone releasing hormone, LHRH); AA =  oral antiandrogens; RP =  Radical prostatectomy/Curative Treatment; RT =  radiation therapy; WW/AS =  watchful waiting(WW)/active surveillance (AS); HRs =  Hazard Ratios; RRs =  Risk Ratios; AMI =  Acute Myocardial Infarction; SD =  standard deviation; NA =  not applicable; CaPSURE  =  Cancer of the Prostate Strategic Urologic Research Endeavor; ICES =  Institute for Clinical Evaluative Sciences; NPCR =  National Prostate Cancer Register; SEER =  Surveillance, Epidemiology and End Results Medicare data; GPRD =  UK General Practice Research Database.

a mean or median.

b compared with WW/AS.

c The HR/RR was directly given in the publication.

d Combined estimates from all types of ADT with random effects meta-analysis.

### Association between ADT and CVD

**Figure 2:**
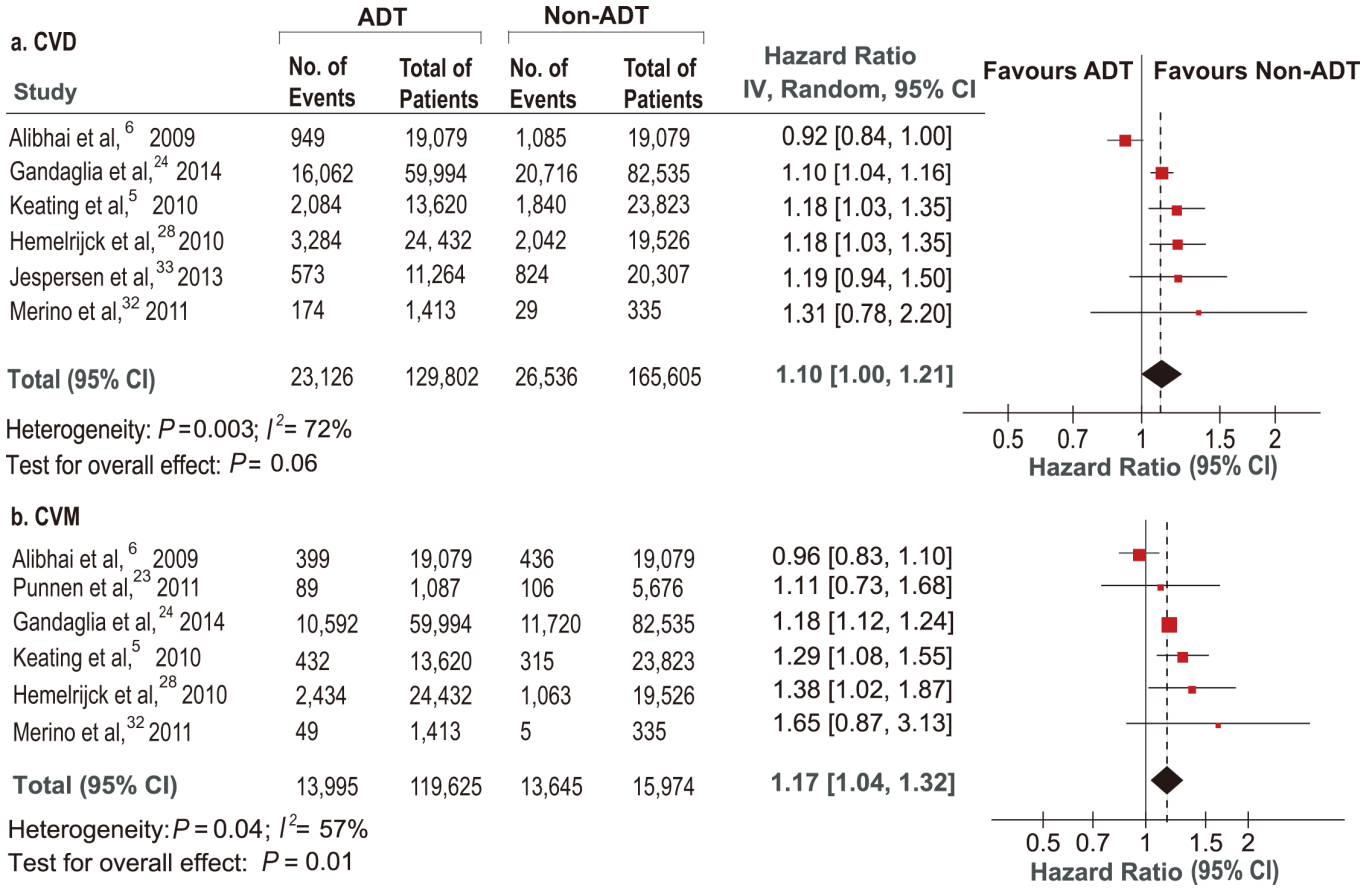
HRs of Cardiovascular Events Related to ADT.

Six studies [5], [6], [24], [28], [32], [33] were identified to investigate the relationship between ADT and CVD. Data from five studies [5], [24], [28], [32], [33] were available for subgroup-analyses comparing different types of ADT with control: three [5], [28], [32] were available for AA and GnRH plus AA, four [5], [24], [28], [32] for GnRH and five [5], [24], [28], [32], [33] for orchiectomy. Among 129,802 ADT users, 23,126 (17.8%) developed CVD compared with 26,536 events (16.0%) among 165,605 non-ADT users (HR = 1.10; 95% CI, 1.00–1.21; *P* = 0.06; Figure 2a). As shown in Figure S1a in File S1, subgroup analyses for different types of ADT indicated that CVD was related with both GnRH (HR = 1.19; 95% CI 1.04–1.36; *P* = 0.010) and GnRH plus AA (HR = 1.46; 95% CI 1.03–2.08; *P* = 0.04), but not with AA alone (HR = 0.94; 95% CI 0.85–1.03; *P* = 0.16) or orchiectomy (HR = 1.15; 95% CI 0.92–1.43; *P* = 0.23). Details of meta-analyses for each type of ADT were shown in Figure S2 in File S1.

### Association between ADT and CVM

Six studies [5], [6], [23], [24], [28], [32] involving 119,625 ADT users and 150,974 controls were identified to analyze the risk of CVM related to ADT. Four studies [5], [24], [28], [32] were available for subgroup-analyses comparing different types of ADT with control: three [5], [28], [32] were available for AA and GnRH plus AA; four [5], [24], [28], [32] for GnRH and orchiectomy. A total of 13,995 CVM events happened in ADT group, accounting for 11.7% of ADT users. In control group, there were 13,645 CVM events, with the overall incidence of 9.0%. Overall analysis showed that ADT was significantly associated with CVM (HR = 1.17; 95% CI, 1.04–1.32; *P* = 0.01; Figure 2b). Subgroup-analyses for different types of ADT were also addressed. Significantly increased risks of CVM were observed in both GnRH (HR = 1.36; 95% CI 1.10–1.68; *P* = 0.004), GnRH plus AA groups (HR = 1.44; 95% CI 1.33–1.57; *P<*0.001), and orchiectomy group (HR = 1.69; 95% CI 1.06–2.71; *P* = 0.03). Non-significantly increased CVM risk was detected in AA alone (HR = 0.95; 95% CI 0.70–1.27; *P = *0.72; Figure S1b in File S1). Details of subgroup-analyses were shown in Figure S3 in File S1.

### ADT Monotherapy vs WW/AS for CVD and CVM

**Figure 3:**
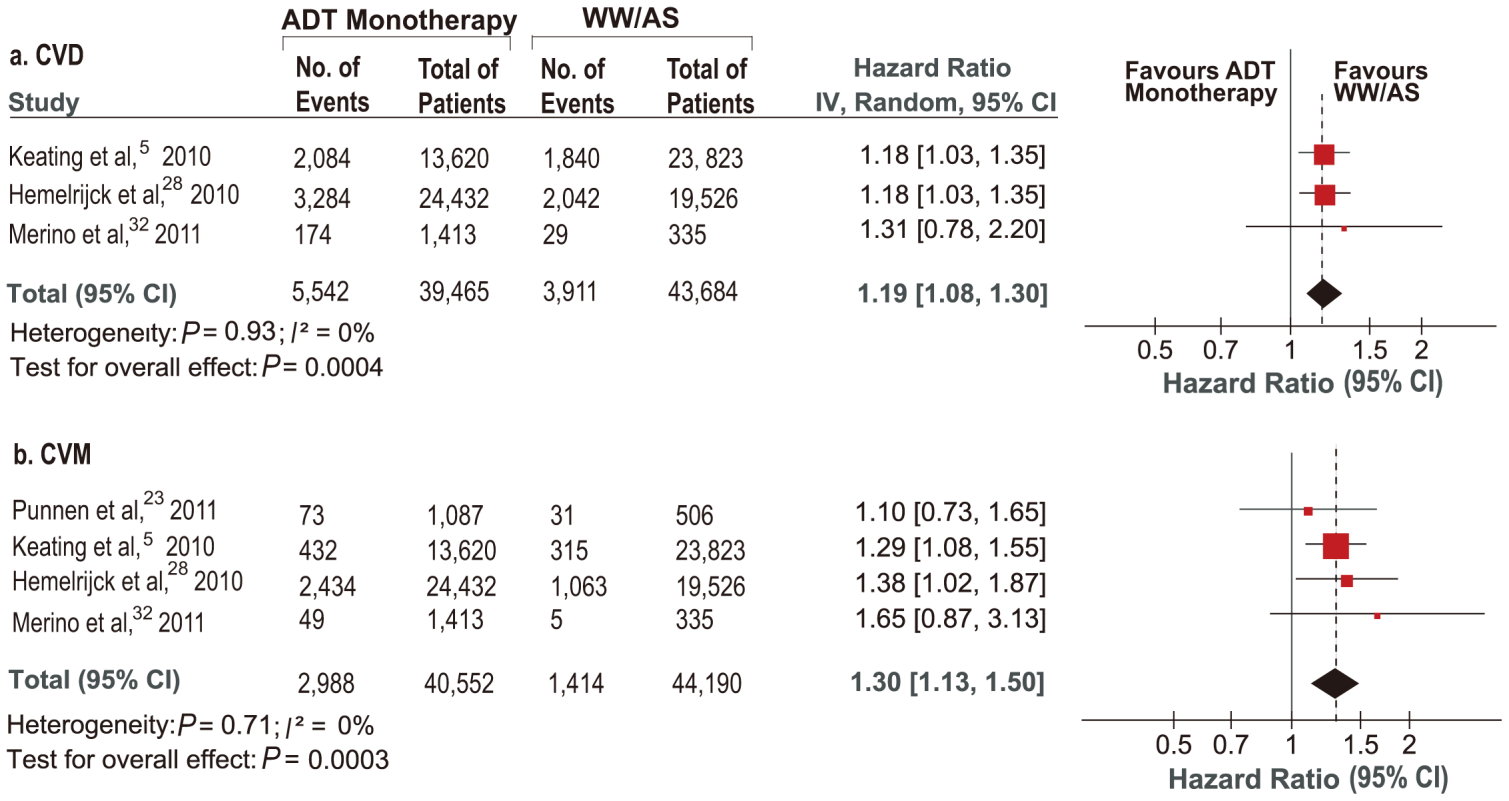
HRs of Cardiovascular Events Related to ADT Monotherapy vs WW/AS.

Treatment of radiotherapy or prostatectomy was used in both ADT and control groups in some studies [23], [28]. In order to minimize the influence of prior treatments, we attempted to exclude patients receiving radiotherapy or prostatectomy. Then comparison of ADT monotherapy vs WW/AS was addressed. Three [5], [28], [32] were included for outcome of CVD. A total of 9,453 events were recorded, containing 5,542 from 39,465 ADT users and 3,911 from 43,684 WW/AS groups. Pooled result revealed that ADT monotherapy significantly increased the risk of CVD (HR = 1.19, 95%CI: 1.08–1.30, *P* = 0.0004; Figure 3a). Four [5], [23], [28], [32] were included in the analysis for CVM. Among 40,552 ADT users, 2,988 developed CVM compared with 1,414 among 44,190 patients with WW/AS. Pooled data showed ADT was significantly associated with CVM, and the incidence was 30% higher than WW/AS (HR = 1.30, 95%CI: 1.13–1.50, *P* = <0.001, Figure 3b).

### Association between ADT and AMI

Definitions of CVD in eligible studies were not identical: some [32], [33] only investigated acute myocardial infarction (AMI), but some others [5], [28] focused on ischemic heart disease including AMI. In order to reduce the influence of inconsistent endpoints on our conclusion, comparison of AMI related to ADT vs control was performed. Six studies [5], [6], [24], [28], [32], [33] with 129,802 ADT users and 165,605 controls were identified. Details of the included studies are summarized in Table S3 in File S1. The available evidence indicated that ADT had a positive but not statistically significant adverse effect on AMI (HR: 1.10, 95%CI, 0.97–1.26; *P* = 0.14; Figure S4 in File S1). As to subgroup analyses for different types of ADT, we noted a positive association between GnRH and AMI (HR = 1.20, 95%CI, 1.05–1.38; *P* = 0.008). On the contrary, treatment with AA alone could even reduce the incidence of AMI (HR = 0.88, 95%CI, 0.81–0.96; *P* = 0.002; Figure S5 in File S1).

### Publication Bias

In our assessment of publication bias, funnel plots showed balance, with points distributing around the verticals, indicating no obvious publication bias (Figure S6 in File S1). Additionally, actualized data from Begg's and Egger's tests also suppoted no exhibited publication bias (Table S4 in File S1).

## Discussion

Although data have accumulated over recent years, the clinical issue weather ADT increases the risk of cardiovascular events remains uncertain. The previous meta-analysis [7] based on 8 RCTs, published on the same topic as ours, collected information from a population of 4,141 patients with local PCa and found a non-significant result with trend benefited ADT. However, our meta-analysis incorporating 6 large-scale observational studies with 295,407 participants showed that ADT was associated with statistically increased CVM and a tendency to increase the risk of CVD. The direct evidence was provided by Tsai et al [22], reporting a significantly increased risk of CVM over a median follow-up of 3.8 years in ADT users with PCa (adjusted HR, 2.6, 95% CI, 1.4–4.7). Another study [4] with 22,816 patients showed a 20% higher risk of CVD in newly diagnosed PCa patients who received ADT for at least 1 year compared with similar men who never received ADT.

According to the 2012 National Comprehensive Cancer Network guidelines [34], the aim of ADT is to reduce serum testosterone to the recommended levels as low as 50 ng/dl. However, serum testosterone deficiency is associated with numerous cardiovascular risk factors, such as increased levels of triglycerides, low-density lipoprotein cholesterol, proinflammatory factors, thickness of the arterial wall and endothelial dysfunction [35]–[38]. Additionally, previous researches [39], [40] showed that deficiency of testosterone could increase body fat mass, hypertension and procoagulant state, as well as fibrinogen and plasminogen activator inhibitor type 1 activity [41]. Studies from animal models of atherosclerosis have showed that aortic atherosclerosis was increased after castration, which is an effect inhibited by testosterone [42], [43]. Basic researches have demonstrated deposited lipid in aortic root of androgen-deficient models, which is the first stage of atherosclerotic plaque development [44]. All of these listed above supported our finding that ADT is a risk factor for cardiovascular events. In addition to cardiovascular disease, ADT is also associated with various side effects such as diabetes, obesity, metabolic syndrome, sarcopenia, osteoporosis, and fracture [45], [46].

Among included studies, one [6] with patients over the age of 66 years who underwent continuous ADT for at least 6 months or bilateral orchiectomy was distinctly discordant with our findings, showing that ADT was not associated with increased risk of AMI (HR = 0.91; 95%CI, 0.84–1.00) or CVM (HR = 0.96; 95% CI, 0.83–1.10). The inconsistency was likely due to the prior treatment received by some patients in both ADT and control groups. Inclusion criteria of patients (>66 years) and long duration of ADT (≥6 months) may also affect the result to some extent. To reduce the intervention bias, we performed the subgroup analysis comparing ADT monotherapy with WW/AS for cardiovascular events. When ADT users received previous treatments were ruled out of consideration, more significantly increased risk of CVD and CVM were found in men treated with ADT monotherapy.

Because of varied types of ADT used in different eligible studies, bias may exist in the results of overall-analyses. In order to reduce the heterogeneities, subgroup analyses on different types of ADT vs control for CVD and CVM were conducted. Significantly increased risk of CVD and CVM were associated with both treatments of GnRH and GnRH plus AA, but not with AA alone or prostatectomy. GnRH agonist could be responsible for cardiovascular toxicity not only through indirect mechanism, in which hypogonadism plays a critical role in the onset of metabolic syndrome, but also through the direct mechanism due to possible presence of GnRH receptors on the heart leading to lower cardiac contractility [47]. The direct evidence provided by Keating et al. [5], evaluating the relationship between GnRH agonist and newly diagnosed CVD, was in accordance with our findings that GnRH agonist could significantly increase CVD (adjusted HR = 1.21, 95%CI:1.06–1.39) and CVM (adjusted HR = 1.28, 95%CI:1.05–1.57).

As to the meta-analysis based on RCTs, one lately published trail [48] reporting cardiac death related to ADT was updated. However, the outcomes were scarcely affected (RR = 0.93; 95%CI, 0.79–1.09; *P* = 0.36; Figure S7 in File S1). Compared with the meta-analysis from RCTs [7], this meta-analysis based on observational studies has a number of strengths. First, we only included population-based observational studies from national-wide databases with long duration of follow-up. Second, the contamination bias is likely to have been minimized because all of the patients included in control groups had never received ADT. Third, all the data available to calculate HRs or RRs were adjusted for the different durations of follow-up in all these included studies if they were not directly given. In addition, for purpose of investigating rare adverse drug reactions, the credibility is much higher to perform a meta-analysis of large-scale observational studies with long duration of follow-up, high quality of design and implementation [14]. All of these listed above made the results of our meta-analysis more credible.

However, some inherent limitations in this meta-analysis should be taken into consideration. Firstly, as in almost all of the meta-analyses, results may be influenced by selection bias. In our study, predesigned search strategy was performed to minimize selection bias as extensive as we could, with independent selection and data extraction by 2 separate reviewers. Studies were included without any language restriction and including registries. Secondly, incomplete data in some included studies may influence the overall result of our study to some extent. HRs or RRs were not directly given in some papers [28], [32], we had to calculate them through the number of cardiovascular events in both ADT and control groups as a result; when two or more types of ADT groups from one study were respectively compared with the same control group (e.g. AA alone vs control, GnRH alone vs control), we used random effect meta-analysis to combine these different data together for compositing overall result. Thirdly, the diversity of cardiovascular events definitions in different studies may affect the results of overall analyses. In order to reduce the bias, confirmatory subgroup analysis for AMI risk was conducted. Fourthly, the certain characteristics of patients (e.g. age, pathologic stages, comorbidities, ADT duration etc.) that may contribute to cardiovascular events were different in each included study, which might substantially confound the presented results. So, adjusted data were extracted when available to minimize the bias. Furthermore, a proportion of those patients included in some studies [23], [28], [33] were diagnosed with metastatic PCa, while some others were local or regional disease, which may impact the interpretation of the results and the significance of the findings. However, the HRs directly given in two publications [23], [33] were already adjusted for the baseline characteristics of patients including prostate cancer stage. Additionally, standardized incidence ratios and standardized mortality ratios were also adjusted for the prostate cancer stage in the other study [28]. Therefore, the influence of mixing populations of prostate cancer stage on our meta-analysis would be minimized.

## Conclusions

In conclusion, pooled result shows that ADT could significantly increase the risk of CVM. Although no significant association is observed between ADT and CVD, there is still a tendency favoring non-ADT users. After removing patients received other treatments, such as prostatectomy and radiotherapy, much stronger associations of ADT with CVD and CVM are observed. Moreover, subgroup analyses for different types of ADT suggest that GnRH and GnRH plus AA, but not AA alone or orchidectomy, can significantly lead to both CVD and CVM. These findings may help clinicians make clinical decision when prescribing ADT. Additional studies are needed to further define populations for whom benefits from ADT outweigh risks and to develop strategies to prevent ADT-related cardiovascular events.

## Supporting Information

File S1 **Containing the following contents.** Methods S1. Literature Search Strategy. Table S1. List of Excluded Full-text Articles with Reasons for Exclusions, for Both CVD and CVM. Table S2. Newcastle-Ottawa Scale Quality Assessment of Included Studies, for both CVD and CVM. Table S3. Characteristics of Studies Investigating AMI Related to ADT. Table S4. Pooled Results and Publication Bias for All Comparisons. Figure S1. HRs of Subgroup Analyses for CVD and CVM Related to Different Types of ADT. Figure S2. Details of Subgroup Analyses for CVD Related to Different Types of ADT. Figure S3. Details of Subgroup Analyses for CVM Related to Different Types of ADT. Figure S4. HR of AMI Morbidity Related to ADT. Figure S5. Details of Subgroup Analyses for AMI Related to Different Types of ADT. Figure S6. Funnel Plots for All Meta-analyses. Figure S7. Updated Meta-analysis from RCTs for CVM related to ADT.
(DOC)

Checklist S1 **PRISMA checklist.** Preferred Reporting Items for Systematic Reviews and Meta-Analyses.
(DOC)

Checklist S2 **MOOSE checklist.** Meta-analysis Of Observational Studies in Epidemiology.
(DOC)
