# Supplementary material for: Androgen Deprivation Therapy for Prostate Cancer Is Associated with Cardiovascular Morbidity and Mortality: A Meta-Analysis of Population-Based Observational Studies
Source: PLoS One. 2014 Sep 29;9(9):e107516. doi: 10.1371/journal.pone.0107516 (PMC4180271; doi:10.1371/journal.pone.0107516)

**Methods S1:** **Literature Search Strategy**

Search date: May 31^th^ 2014

Databases searched: MEDLINE, EMBASE, and Cochrane Library Central Register.

**1. Database: MEDLINE**

Search Strategy:

| #1 | Search prostate cancer [MeSH] OR prostate tumor [MeSH] OR prostate carcinoma [MeSH] Field: Full text | 121,349 |
| --- | --- | --- |
| #2 | Search androgen deprivation [MeSH] OR androgen suppression [MeSH] OR endocrine treatment [MeSH] OR ADT [MeSH] OR AST [MeSH] Field: Full text | 172,366 |
| #3 | Search #1 AND #2 Field: Full text | 7,444 |
| #4 | Search cardiovascular [MeSH] OR myocardial infarction [MeSH] OR coronary heart disease [MeSH] OR cardiac death [MeSH] OR AMI [MeSH] Field: Full text | 2080,148 |
| #5 | Search #3 AND #4 Field: Full text | 873 |

**2. Database: EMBASE**

**Search Strategy**:

| #1 | **'prostate'**/exp AND **'cancer'**/exp OR **'prostate'**/exp AND **'tumor'**/exp OR **'prostate'**/exp AND **'carcinoma'**/exp AND [humans]/lim AND[abstracts]/lim | 3,056 |
| --- | --- | --- |
| #2 | **'androgen'**/exp AND **deprivation** OR (**'androgen'**/exp AND **suppression**) OR (**endocrine** AND **treatment**)OR**ADT** OR **AST** AND[humans]/limAND[full text]/lim | 801,123 |
| #3 | #1 AND #2 Not animals full text. | 389 |
| #4 | **cardiovascular** OR **myocardial** AND **'infarction'**/expOR **coronary** AND  **'heart'**/expAND **'disease'**/expOR **ischemic** AND **'heart'**/expAND**'disease'**/exp OR **cardiac** AND **'death'**/exp OR **AMI** AND [humans]/lim AND [full text]/lim | 35,163 |
| #5 | #3 AND #4 full text. Not animals | 138 |

**3. Database: The Cochrane Library Central Register**

Search Strategy:

| #1 | prostate cancer or prostate tumor or prostate carcinoma: all text, kw (Word variations have been searched) | 6,458 |
| --- | --- | --- |
| #2 | androgen deprivation or androgen suppression or endocrine treatment or ADT or AST: all text kw (Word variations have been searched) | 5,264 |
| #3 | #1 AND #2 all text | 670 |
| #4 | cardiovascular or myocardial infarction or coronary heart disease or cardiac death or AMI all text kw (Word variations have been searched) | 58,838 |
| #5 | #3 AND #4 all text | 94 |

**Table S1.** **List of Excluded Full-text Articles with Reasons for Exclusions, for Both CVD and CVM**

|  |  | **Reason for exclusion** |
| --- | --- | --- |
| 1 | Efstathiou JA, Bae K, Shipley WU, Hanks GE, Pilepich MV, Sandler HM, et al. Cardiovascular mortality and duration of androgen deprivation for locally advanced prostate cancer: analysis of RTOG 92-02. European urology. 2008;54(4):816-23. | ADT was also used in control group; study design was RCT |
| 2 | Soloway MS, Hachiya T, Ruiz HE, Gomez CC, Civantos F. Significance of androgen deprivation prior to radical prostatectomy, with special reference to prostate-specific antigen. World journal of urology. 1993;11(4):221-6. | ADT was also used in control group |
| 3 | Hayes JH, Chen MH, Moran BJ, Braccioforte MH, Dosoretz DE, Salenius S, et al. Androgen-suppression therapy for prostate cancer and the risk of death in men with a history of myocardial infarction or stroke. BJU international. 2010;106(7):979-85. | Cardiovascular events were not the end point |
| 4 | Van Hemelrijck M, Garmo H, Holmberg L, Stattin P, Adolfsson J. Multiple events of fractures and cardiovascular and thromboembolic disease following prostate cancer diagnosis: results from the population-based PCBaSe Sweden. European urology. 2012;61(4):690-700. | Duplicate – completed with  Hemelrijck et al. 2010 |
| 5 | Robinson D, Garmo H, Lindahl B, Van Hemelrijck M, Adolfsson J, Bratt O, et al. Ischemic heart disease and stroke before and during endocrine treatment for prostate cancer in PCBaSe Sweden. International journal of cancer Journal international du cancer. 2012;130(2):478-87. | Duplicate – completed with  Hemelrijck et al. 2010 |
| 6 | Hedlund PO, Ala-Opas M, Brekkan E, Damber JE, Damber L, Hagerman I, et al. Parenteral estrogen versus combined androgen deprivation in the treatment of metastatic prostatic cancer -- Scandinavian Prostatic Cancer Group (SPCG) Study No. 5. Scandinavian journal of urology and nephrology. 2002;36(6):405-13. | ADT was also used in control group |
| 7 | Hedlund PO, Damber JE, Hagerman I, Haukaas S, Henriksson P, Iversen P, et al. Parenteral estrogen versus combined androgen deprivation in the treatment of metastatic prostatic cancer: part 2. Final evaluation of the Scandinavian Prostatic Cancer Group (SPCG) Study No. 5. Scandinavian journal of urology and nephrology. 2008;42(3):220-9. | ADT was also used in control group |
| 8 | Taira AV, Merrick GS, Galbreath RW, Butler WM, Wallner KE. Factors impacting all-cause mortality in prostate cancer brachytherapy patients with or without androgen deprivation therapy. Brachytherapy. 2010;9(1):42-9. | Data is not comprehensive: data of CV mortality was only available in low- testosterone patients |
| 9 | Messing EM, Manola J, Yao J, Kiernan M, Crawford D, Wilding G, et al. Immediate versus deferred androgen deprivation treatment in patients with node-positive prostate cancer after radical prostatectomy and pelvic lymphadenectomy. The lancet oncology. 2006;7 (6):472-9. | ADT was also used in control group; study design was RCT |
| 10 | Mikkola A, Aro J, Rannikko S, Oksanen H, Ruutu M. Cardiovascular complications in patients with advanced prostatic cancer treated by means of orchiectomy or polyestradiol phosphate. Scandinavian journal of urology and nephrology. 2005;39(4):294-300. | ADT was also used in control group |
| 11 | Studer UE, Whelan P, Albrecht W, Casselman J, de Reijke T, Hauri D, et al. Immediate or deferred androgen deprivation for patients with prostate cancer not suitable for local treatment with curative intent: European Organisation for Research and Treatment of Cancer (EORTC) Trial 30891. Journal of clinical oncology : official journal of the American Society of Clinical Oncology. 2006;24(12):1868-76. | ADT was also used in control group; study design was RCT |
| 12 | Schroder FH, Kurth KH, Fossa SD, Hoekstra W, Karthaus PP, De Prijck L, et al. Early versus delayed endocrine treatment of T2-T3 pN1-3 M0 prostate cancer without local treatment of the primary tumour: final results of European Organisation for the Research and Treatment of Cancer protocol 30846 after 13 years of follow-up (a randomised controlled trial). European urology. 2009;55(1):14-22. | ADT was also used in control group; study design was RCT |
| 13 | Denham JW, Steigler A, Lamb DS, Joseph D, Turner S, Matthews J, et al. Short-term neoadjuvant androgen deprivation and radiotherapy for locally advanced prostate cancer: 10-year data from the TROG 96.01 randomised trial. The lancet oncology. 2011;12(5):451-9 | Study design was RCT |
| 14 | Mikkola A, Aro J, Rannikko S, Ruutu M. Ten-year survival and cardiovascular mortality in patients with advanced prostate cancer primarily treated by intramuscular polyestradiol phosphate or orchiectomy. The Prostate. 2007;67(4):447-55 | ADT (Orchiectomy)was also used in control group |
| 15 | Roach M, 3rd, Bae K, Speight J, Wolkov HB, Rubin P, Lee RJ, et al. Short-term neoadjuvant androgen deprivation therapy and external-beam radiotherapy for locally advanced prostate cancer: long-term results of RTOG 8610. Journal of clinical oncology : official journal of the American Society of Clinical Oncology. 2008;26(4):585-91. | Study design was RCT |
| 16 | Bolla M, Van Tienhoven G, Warde P, Dubois JB, Mirimanoff RO, Storme G, et al. External irradiation with or without long-term androgen suppression for prostate cancer with high metastatic risk: 10-year results of an EORTC randomised study. The lancet oncology. 2010; 11 (11): 1066-73. | Study design was RCT |
| 17 | Manikandan R, Srirangam SJ, Pearson E, Brown SC, O'Reilly P, Collins GN. Diethylstilboestrol versus bicalutamide in hormone refractory prostate carcinoma: a prospective randomized trial. Urologia internationalis. 2005;75(3):217-21. | ADT was also used in control group |
| 18 | D'Amico AV, Chen MH, Renshaw AA, Loffredo M, Kantoff PW. Causes of death in men undergoing androgen suppression therapy for newly diagnosed localized or recurrent prostate cancer. Cancer. 2008;113(12):3290-7 | ADT was also used in control group; study design was RCT |
| 19 | Denham JW, Steigler A, Lamb DS, Joseph D, Mameghan H, Turner S, et al. Short-term androgen deprivation and radiotherapy for locally advanced prostate cancer: results from the Trans-Tasman Radiation Oncology Group 96.01 randomised controlled trial. The lancet oncology. 2005;6(11):841-50. | Study design was RCT |
| 20 | D'Amico AV, Denham JW, Crook J, Chen MH, Goldhaber SZ, Lamb DS, et al. Influence of androgen suppression therapy for prostate cancer on the frequency and timing of fatal myocardial infarctions. Journal of clinical oncology: official journal of the American Society of Clinical Oncology. 2007;25(17):2420-5. | Study design was comprised 3 RCTs |
| 21 | Langley RE, Cafferty FH, Alhasso AA, Rosen SD, Sundaram SK, Freeman SC, et al. Cardiovascular outcomes in patients with locally advanced and metastatic prostate cancer treated with luteinizing hormone releasing hormone agonists or transdermal oestrogen: the randomised, phase 2 MRC PATCH trial (PR09). The lancet oncology. 2013;14(4):306-16. | ADT was also used in control group; study design was RCT |
| 22 | Braga-Basaria M, Dobs AS, Muller DC, Carducci MA, John M, Egan J, et al. Metabolic syndrome in men with prostate cancer undergoing long-term androgen-deprivation therapy. Journal of clinical oncology: official journal of the American Society of Clinical Oncology. 2006;24(24):3979-83. | Cardiovascular events were not the end point |
| 23 | D'Amico AV, Chen MH, Renshaw AA, Loffredo M, Kantoff PW. Androgen suppression and radiation vs radiation alone for prostate cancer: a randomized trial. JAMA : the journal of the American Medical Association. 2008;299(3):289-95 | Study design was RCT |
| 24 | Nguyen PL, Chen MH, Goldhaber SZ, Martin NE, Beard CJ, Dosoretz DE, et al. Coronary revascularization and mortality in men with congestive heart failure or prior myocardial infarction who receive androgen deprivation. Cancer. 2011;117(2):406-13 | Cardiovascular events were not the end point |
| 25 | Lage MJ, Barber BL, Markus RA. Association between androgen deprivation therapy and incidence of diabetes among males with prostate cancer. Urology. 2007;70(6):1104-8. | Cardiovascular events were not the end point |
| 26 | Smith MR, Klotz L, van der Meulen E, Colli E, Tanko LB. Gonadotropin releasing hormone blockers and cardiovascular disease risk: analysis of prospective clinical trials of degarelix. The Journal of urology. 2011; 186(5):1835-42. | ADT was also used in control group |
| 27 | Wilcox C, Kautto A, Steigler A, Denham JW. Androgen deprivation therapy for prostate cancer does not increase cardiovascular mortality in the long term. Oncology. 2012;82(1):56-8. | Study design was RCT |
| 28 | Merrick GS, Butler WM, Wallner KE, Galbreath RW, Allen ZA, Adamovich E, Lief J. Androgen deprivation therapy does not impact cause-specific or overall survival in high-risk prostate cancer managed with brachytherapy and supplemental extern. International journal of radiation oncology, biology, physics. 2006;68(1):34-40. | Duplicate – completed with  Merrick et al. 2006 |
| 29 | Cleffi S, Neto AS, Reis LO, Maia P, Fonseca F, Wroclawski ML, et al. Androgen deprivation therapy and morbid obesity: do they share cardiovascular risk through metabolic syndrome?. Actas urologicas espanolas. 2011;35(5):259-65. | Study design was cross-sectional study |
| 30 | Efstathiou JA, Bae K, Shipley WU, Hanks GE, Pilepich MV, Sandler HM, et al. Cardiovascular mortality after androgen deprivation therapy for locally advanced prostate cancer: RTOG 85-31. Journal of clinical oncology : official journal of the American Society of Clinical Oncology. 2009;27(1):92-9. | Study design was RCT |
| 31 | Smith JC, Bennett S, Evans LM, Kynaston HG, Parmar M, Mason MD, et al. The effects of induced hypogonadism on arterial stiffness, body composition, and metabolic parameters in males with prostate cancer. The Journal of clinical endocrinology and metabolism. 2001; 86(9): 4261-7. | ADT was also used in control group |
| 32 | Bhasin S, Jasjua GK, Pencina M, D'Agostino R, Sr., Coviello AD, Vasan RS, et al. Sex hormone-binding globulin, but not testosterone, is associated prospectively and independently with incident metabolic syndrome in men: the framingham heart study. Diabetes care. 2011; 34 (11):2464-70. | Cardiovascular events were not the end point |
| 33 | Bittner N, Merrick GS, Galbreath RW, Butler WM, Wallner KE, Allen ZA, et al. Primary causes of death after permanent prostate brachytherapy. International journal of radiation oncology, biology, physics. 2008; 72(2): 433-40. | Cardiovascular events were not the end point |
| 34 | Nguyen PL, Chen MH, Beckman JA, Beard CJ, Martin NE, Choueiri TK, et al. Influence of androgen deprivation therapy on all-cause mortality in men with high-risk prostate cancer and a history of congestive heart failure or myocardial infarction. International journal of radiation oncology, biology, physics. 2012;82(4):1411-6. | Cardiovascular events were not the end point |
| 35 | Nguyen PL, Chen MH, Hoffman KE, Chen RC, Hu JC, Bennett CL, et al. Cardiovascular comorbidity and treatment regret in men with recurrent prostate cancer. BJU international. 2012;110(2):201-5. | Cardiovascular events were not the end point |
| 36 | Tendulkar RD, Hunter GK, Reddy CA, Stephans KL, Ciezki JP, Abdel-Wahab M, et al. Causes of mortality after dose-escalated radiation therapy and androgen deprivation for high-risk prostate cancer. International journal of radiation oncology, biology, physics. 2013;87(1):94-9. | ADT was also used in control group |
| 37 | Lester-Coll NH, Goldhaber SZ, Sher DJ, D'Amico AV. Death from high-risk prostate cancer versus cardiovascular mortality with hormonal therapy: a decision analysis. Cancer. 2013;119(10):1808-15. | Cardiovascular events were not the end point |
| 38 | Merrick GS, Butler WM, Wallner KE, Galbreath RW, Allen ZA, Adamovich E. Androgen-deprivation therapy does not impact cause-specific or overall survival after permanent prostate brachytherapy. *International journal of radiation oncology, biology, physics.* Jul 1 2006;65(3):669-677. | Database was not population-based |
| 39 | Kim J, Vaid M, Tyldesley S, Woods R, Pickles T. Population-based study of cardiovascular mortality among patients with prostate cancer treated with radical external beam radiation therapy with and without adjuvant androgen deprivation therapy at the British Columbia Cancer Agency. *International journal of radiation oncology, biology, physics.* Jul 1 2011;80(3):742-750. | Study has selection bias (only patients underwent external beam radiation therapy were included). |
| 40 | Huang G, Yeung CY, Lee KK, Liu J, Ho KL, et al. (2014) Androgen deprivation therapy and cardiovascular risk in chinese patients with nonmetastatic carcinoma of prostate. J Oncol 2014: 529468. | Database was not population-based |
| 41 | Keating NL, O'Malley AJ, Smith MR. Diabetes and cardiovascular disease during androgen deprivation therapy for prostate cancer. Journal of clinical oncology : official journal of the American Society of Clinical Oncology. Sep 20 2006;24(27):4448-4456. | Duplicate – completed with  Gandaglia et al. 2014 |

**Table S2. Newcastle-Ottawa Scale Quality Assessment of Included Studies**, for both CVD and CVM

| **study** | **Selection** | | | | | | **Comparability** | **Outcome** | | | **Scores** |
| --- | --- | --- | --- | --- | --- | --- | --- | --- | --- | --- | --- |
|  | Representativeness of exposed cohort | | Selection of non-exposed cohort | | Ascertainment of exposure | outcome of interest was not present at start of study | Comparability on the basis of the design or analysis^a^ | Assessment  of outcome | follow-up long enough for outcomes to occur | Adequacy of  follow up  of cohorts |  |
| Jespersen et al, 2013 | ☆ | ☆ | | ☆ | | ☆ | ☆ | ☆ | ☆ | ☆ | 8 |
| Punnen et al, 2011 | ☆ | ☆ | | ☆ | | ☆ | - | ☆ | ☆ | ☆ | 7 |
| Hemelrijck et al, 2010 | ☆ | ☆ | | ☆ | | ☆ | ☆ | ☆ | ☆ | ☆ | 8 |
| Alibhai et al, 2009 | ☆ | ☆ | | ☆ | | ☆ | ☆☆ | ☆ | ☆ | ☆ | 9 |
| Keating et al, 2010 | - | ☆ | | ☆ | | ☆ | ☆ | ☆ | ☆ | ☆ | 7 |
| Gandaglia et al, 2014 | ☆ | ☆ | | ☆ | | ☆ | ☆ | ☆ | ☆ | ☆ | 8 |
| Merino et al, 2011 | ☆ | ☆ | | ☆ | | ☆ | ☆ | ☆ | ☆ | - | 7 |

^a^ A maximum of 2 stars can be allotted in this category, one for the most important factors (Age) the other for second important factors (gender, race, etc.).

**Table S3. Characteristics of Studies Investigating AMI Related to ADT**

| **First author**  **year** | **Design,**  **LOE** | **Database source**  **(Duration)** | **Definition of**  **AMI (ICD codes)** | **Types of ADT** | **Treatments in control group** | **No. of ADT/Control** | | | **Age**  **y**^a^**(SD)/No. of patients** | **Follow-up, (y**^a^**)** | | **Adjusted HRs（95%CI）** | | |
| --- | --- | --- | --- | --- | --- | --- | --- | --- | --- | --- | --- | --- | --- | --- |
| Jespersen et al  2013 | Cohort,  2a | Danish Cancer Registry  (2002-2010) | AMI (ICD-8 codes 410.09/ 410.99 and IDC-10 codes DI21.x) | GnRH/AA | non-ADT | 9,204 | 20,307 | | 71 | 3.3  (1.8 to 5.2) | | 1.31(1.16,1.49)**^c^** | 1.19 (0.94, 1.50)^d^ | |
|  |  |  |  | Orchiectomy |  | 2,060 |  |  |  |  |  | 0.90(0.83,1.29)**^c^** |  |  |
| Hemelrijck et al  2010 | Cohort,  2a | NPCR of  Sweden  (1997-2007) | AMI  (ICD-10: 121) | GnRH agonist | RP | 9,066 | 26,432 | | ≤65:19153  66 to 74: 27737  ≥75:13110 | 3.8 | 4.4 | 1.34(1.23,1.46)^b^ | 1.21 (1.03, 1.43)^d^ | |
|  |  |  |  | AA |  | 3,391 |  |  |  | 4 |  | 0.89(0.80,0.95)^b^ |  |  |
|  |  |  |  | GnRH+AA | WW/AS | 11,646 | 19,526 | |  | 3.3 | 4.7 | 1.21(1.12,1.32)^b^ |  |  |
|  |  |  |  | Orchiectomy |  | 5,340 |  |  |  | 3.1 |  | 1.45(1.31,1.60)^b^ |  |  |
|  |  |  |  | Other types |  | 1,199 |  |  |  | / |  | / |  |  |
| Merino et al  2011 | Nested Case-  Control.  2a | GPRD  (1999-2005) | hospitalization  from AMI (NA) | GnRH agonist | WW/AS | 572 | 330 | | 51 to 69: 324  70 to 84: 850 | NA | | 1.17(0.82,1.66) | 1.21 (0.57, 2.56)^d^ | |
|  |  |  |  | AA |  | 465 |  |  |  |  |  | 1.02(0.70,1.48) |  |  |
|  |  |  |  | GnRH+AA |  | 30 |  |  |  |  |  | 2.41(1.30,4.48) |  |  |
|  |  |  |  | Orchiectomy |  | 9 |  |  |  |  |  | 0.89(0.14,5.80) |  |  |
| Alibhai et al  2009 | Cohort,  2a | ICES  (1995-2005) | AMI (ICD-9-CM 410.0-410.9) | ADT | WW/AS | 19,079/19,079 | | | 75±6.3 | 6.47 | | 0.92(0.84,1.00)**^c^** | | |
| Keating et al  2010 | Cohort,  2a | Veterans Healthcare Administration  (2001-2004) | AMI (ICD-9 codes 410.XX except 410.X2) | GnRH agonist | WW/AS | 13,065 | | 23,823 | 66.9±8.6 | 2.6 | | 1.21(1.01,1.44)**^c^** | | 1.24 (0.97, 1.58)^d^ |
|  |  |  |  | AA |  | 1,230 | |  |  |  |  | 0.98(0.43,2.19)**^c^** | |  |
|  |  |  |  | GnRH+AA |  | 1,829 | |  |  |  |  | 0.99(0.59,1.64)**^c^** | |  |
|  |  |  |  | Orchiectomy |  | 268 | |  |  |  |  | 1.98(1.15,3.41)**^c^** | |  |
| Gandaglia et al,2014 | Cohort,  2a | SEER  (1995-2009) | AMI (ICD-9) | GnRH agonist | non-ADT | 57,939 | | 82,535 | 73.6 (69 to 77) | 6.28 | | 1.09(1.04,1.15)**^c^** | | 1.03 (0.89,1.20)^d^ |
|  |  |  |  | Orchiectomy |  | 2,055 | |  |  |  |  | 0.99(0.83,1.20)**^c^** | |  |

Abbreviations: LOE=level of evidence; AMI= Acute Myocardial Infarction; ADT=androgen deprivation therapy; GnRH=gonadotropin-releasing hormone (leuteinizing hormone releasing hormone, LHRH); AA=oral antiandrogens; RP=Radical prostatectomy/Curative Treatment; WW/AS=watchful waiting (WW)/active surveillance (AS); HRs=Hazard Ratios; SD=standard deviation; NA=not applicable; NPCR=National Prostate Cancer Register; SEER=Surveillance, Epidemiology and End Results Medicare data; GPRD=UK General Practice Research Database; ICES=Institute for Clinical Evaluative Sciences.

^a^ mean or median

^b^ compared with WW/AS

^c^ The HR was directly given in the publication

^d^ Combined estimates from all types of ADT with random effects meta-analysis

**Table S**4. Pooled Results and Publication Bias for All Comparisons

| **Measurement** | **n^b^** | **Case/control** | **Heterogeneity** | | **Pooled rate/HR**  **(95% CI)** | **Begg’s test (*P*)** | **Egger’s test (*P*)** |
| --- | --- | --- | --- | --- | --- | --- | --- |
|  |  |  | ***P*** | ***I^2^* (%)** |  |  |  |
| ***CVD*** |  |  |  |  |  |  |  |
| ADT vs Non-ADT | 6 | 129,802/165,605 | 0.003 | 72 | **1.10 (1.00-1.21)** | 1.000 | 0.149 |
| AA vs Non-ADT | 3 | 5,002/43,684 | 0.430 | 0 | 0.94 (0.85-1.03) | 0.602 | 0.778 |
| GnRH vs Non-ADT | 4 | 89,865/126,219 | <0.001 | 86 | **1.19 (1.04-1.36)** | 1.000 | 0.731 |
| GnRH plus AA vs Non-ADT | 3 | 13,906/43,684 | 0.006 | 81 | **1.46 (1.03-2.08)** | 0.296 | 0.298 |
| Orchiectomy vs Non-ADT | 5 | 9,733/146,526 | <0.001 | 81 | 1.15 (0.92-1.43) | 1.000 | 0.884 |
| ADT vs WW/AS^a^ | 3 | 39,465/43,684 | 0.930 | 0 | **1.19 (1.08-1.30)** | 0.734 | 0.699 |
| ***CVM*** |  |  |  |  |  |  |  |
| ADT vs Non-ADT | 6 | 119,625/150,974 | 0.040 | 57 | **1.17 (1.04-1.32)** | 0.707 | 0.246 |
| AA vs Non-ADT | 3 | 5,012/70,116 | 0.001 | 86 | 0.95 (0.70-1.27) | 0.602 | 0.769 |
| GnRH vs Non-ADT | 4 | 80,661/152,651 | <0.001 | 91 | **1.36 (1.10-1.68)** | 1.000 | 0.935 |
| GnRH plus AA vs Non-ADT | 3 | 13,915/70,116 | 0.460 | 0 | **1.44 (1.33-1.57)** | 1.000 | 0.870 |
| Orchiectomy vs Non-ADT | 4 | 7,673/152,651 | <0.001 | 91 | **1.69 (1.06-2.71)** | 1.000 | 0.885 |
| ADT vs WW/AS^a^ | 4 | 40,552/44,190 | 0.710 | 0 | **1.30 (1.13-1.50)** | 1.000 | 0.240 |
| ***AMI Morbidity*** |  |  |  |  |  |  |  |
| ADT vs Non-ADT | 6 | 129,802/165,605 | 0.008 | 68 | 1.10 (0.97-1.26) | 0.452 | 0.108 |
| AA vs Non-ADT | 3 | 5,086/43,678 | 0.710 | 0 | **0.88 (0.81-0.96)** | 0.602 | 0.363 |
| GnRH vs Non-ADT | 4 | 80,642/126,213 | 0.001 | 82 | **1.20 (1.05-1.38)** | 0.734 | 0.863 |
| GnRH plus AA vs Non-ADT | 3 | 13,505/43,678 | 0.070 | 63 | 1.33 (0.92-1.94) | 0.662 | 0.728 |
| Orchiectomy vs Non-ADT | 5 | 9,732/146,520 | <0.001 | 79 | 1.23 (0.96-1.58) | 1.000 | 0.827 |
| Abbreviations: CVD= Cardiovascular disease; CVM= Cardiovascular mortality; AMI= Acute myocardial infarction; ADT= Androgen deprivation therapy; GnRH= gonadotropin-releasing hormone (leuteinizing hormone releasing hormone, LHRH); AA=oral antiandrogens.  ^a^ ADT monotherapy vs WW/AS  ^b^ Number of included studies | | | | | | | |

**Figure S1. HRs of Subgroup Analyses for CVD and CVM Related to Different Types of ADT**


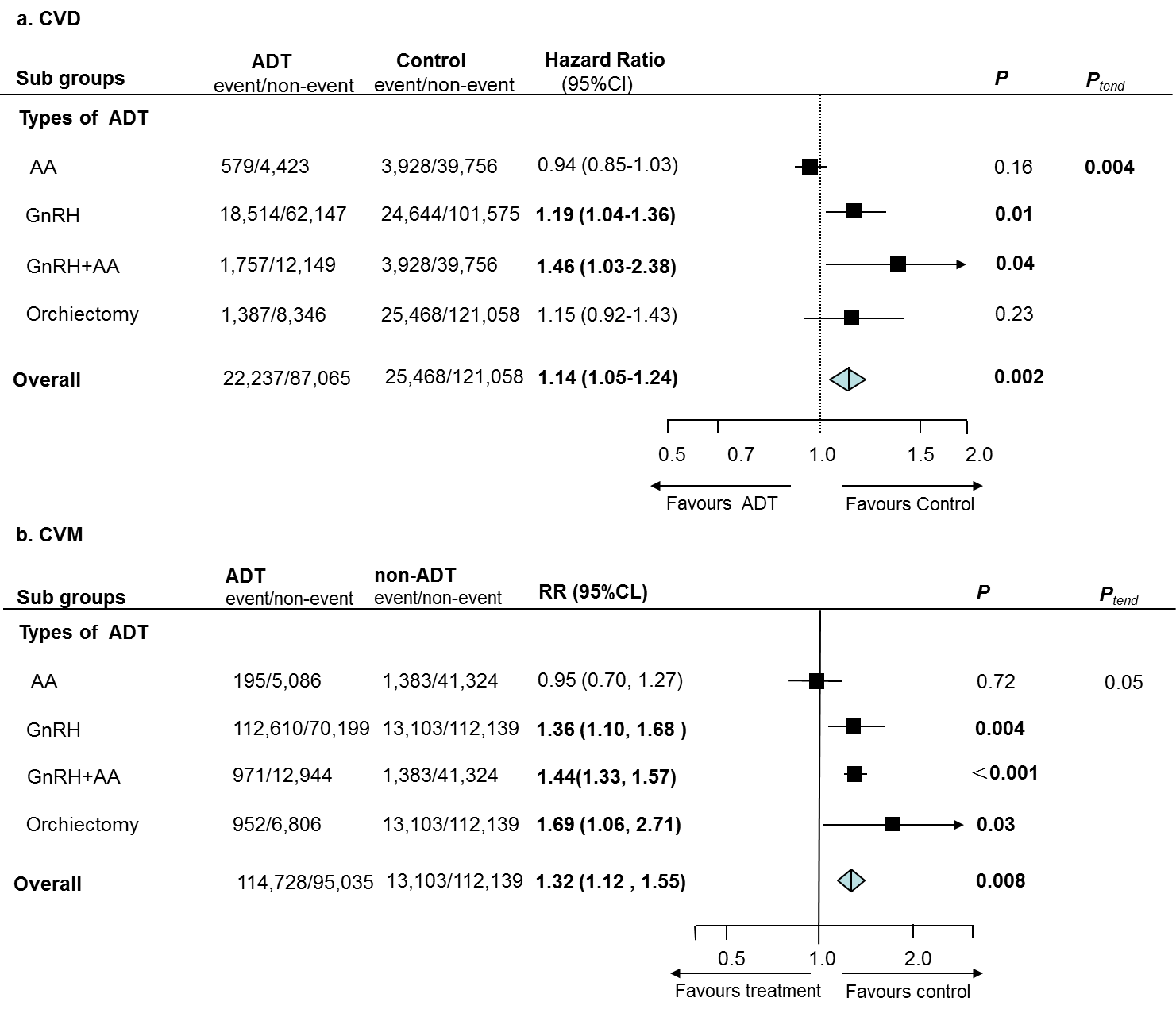


**Figure S2. Details of Subgroup Analyses for CVD Related to Different Types of ADT**


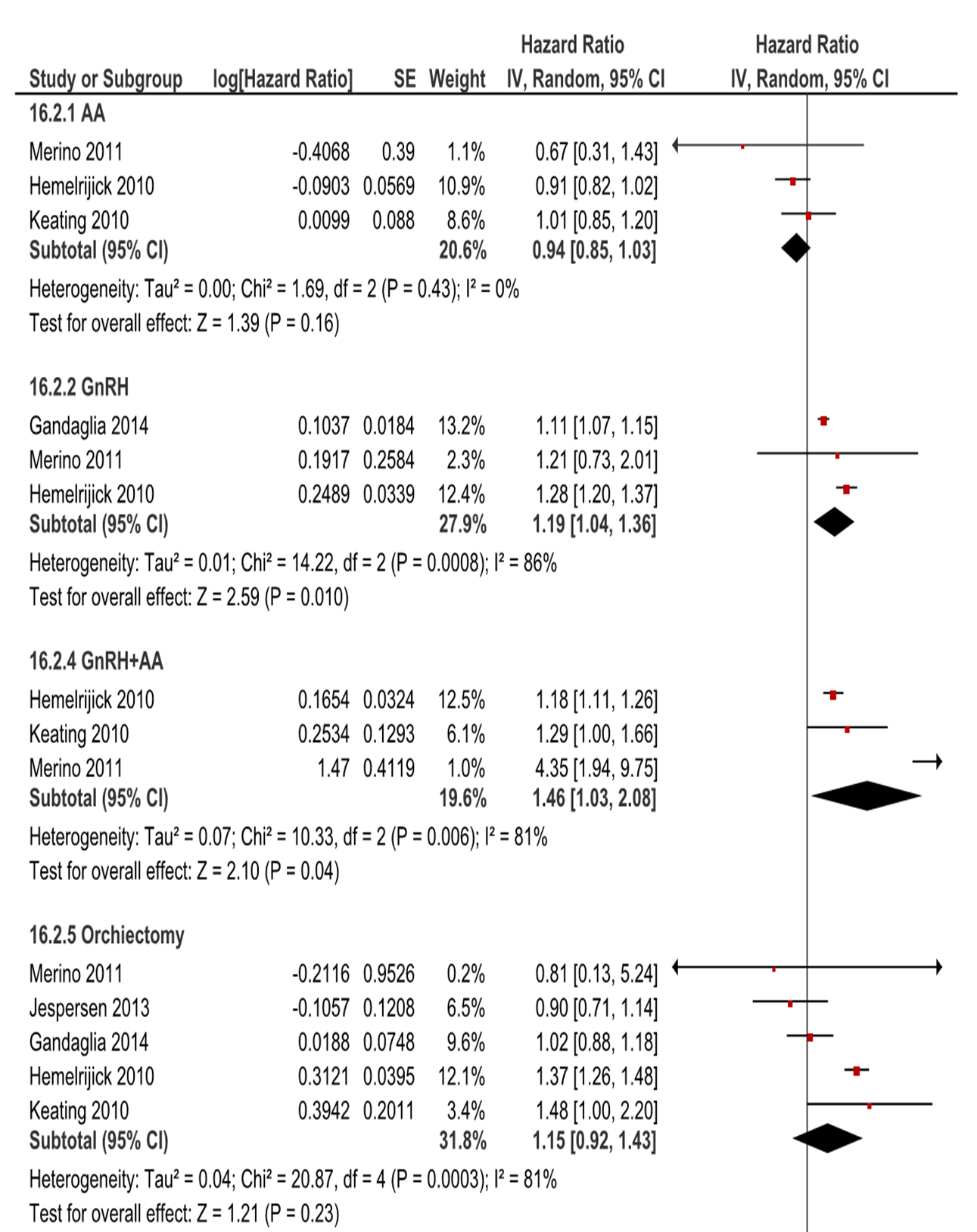


**Figure S3. Details of Subgroup Analyses for CVM Related to Different Types of ADT**


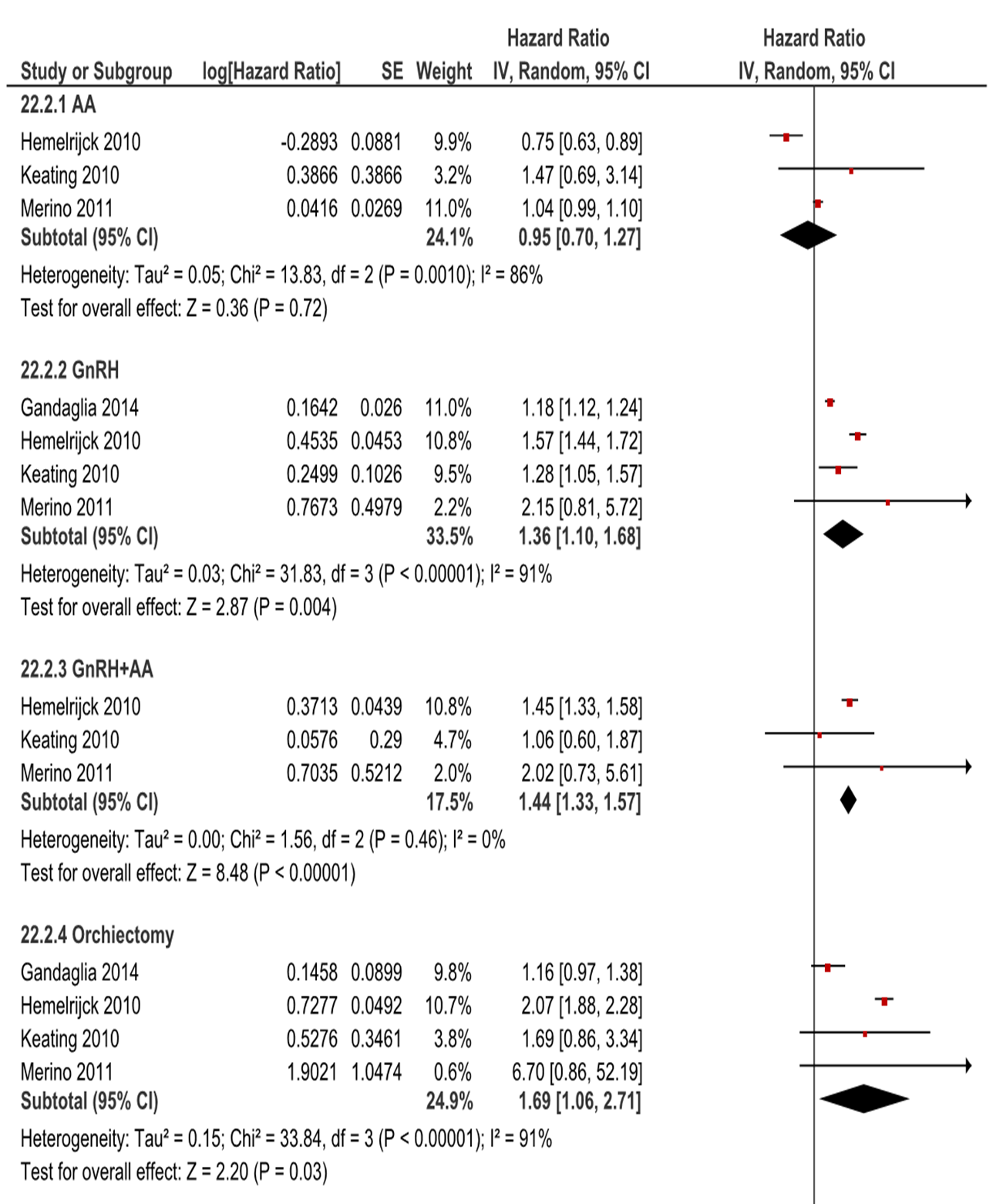


**Figure S4. HR of AMI Morbidity Related to ADT**


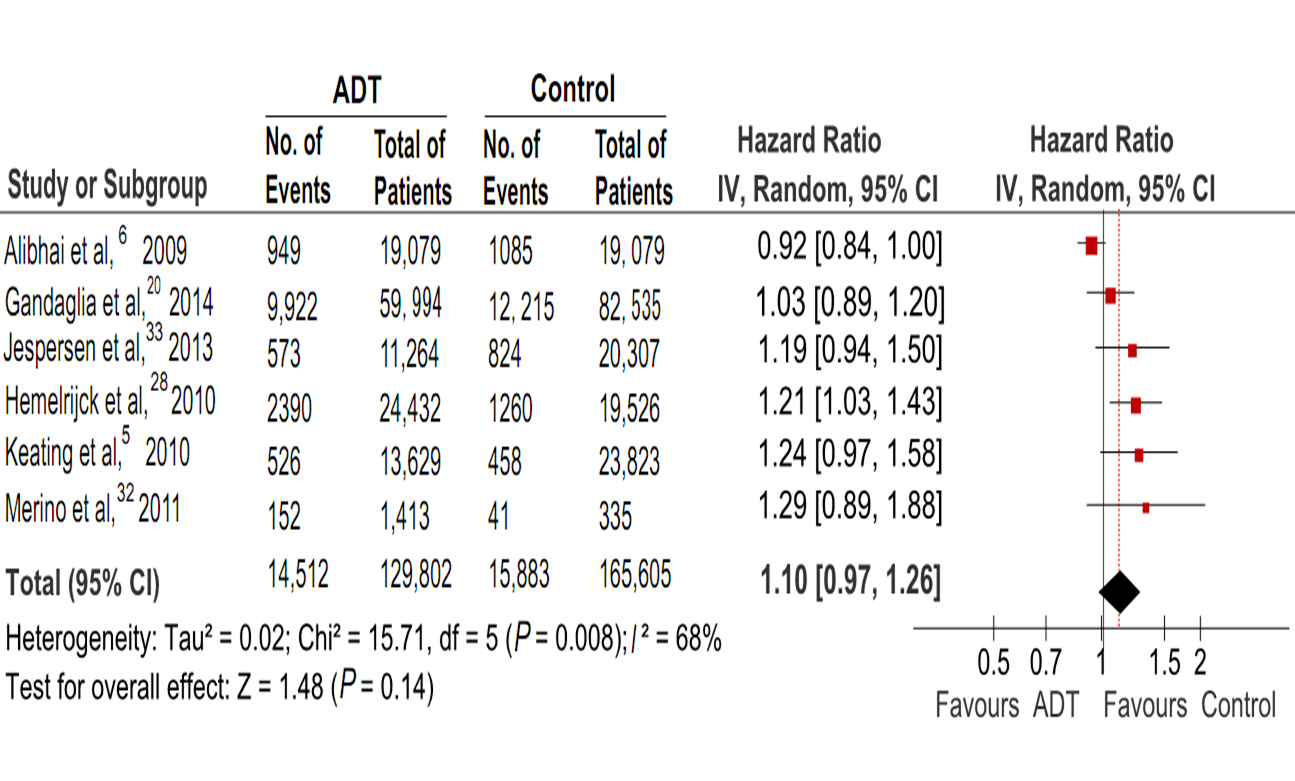


**Figure S5. Details of Subgroup Analyses for AMI Related to Different Types of ADT**


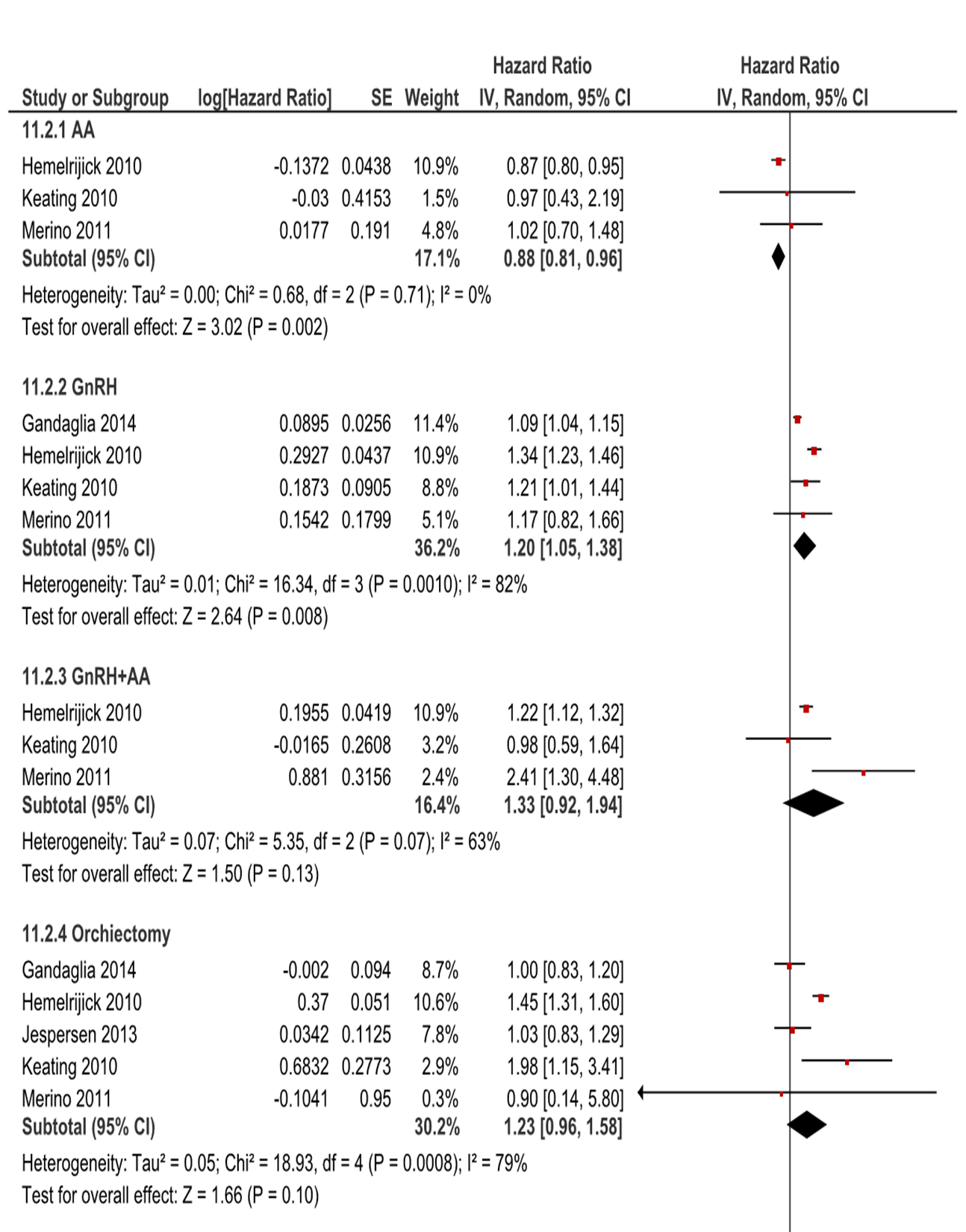


**Figure S6: Funnel Plots for All Meta-analyses**


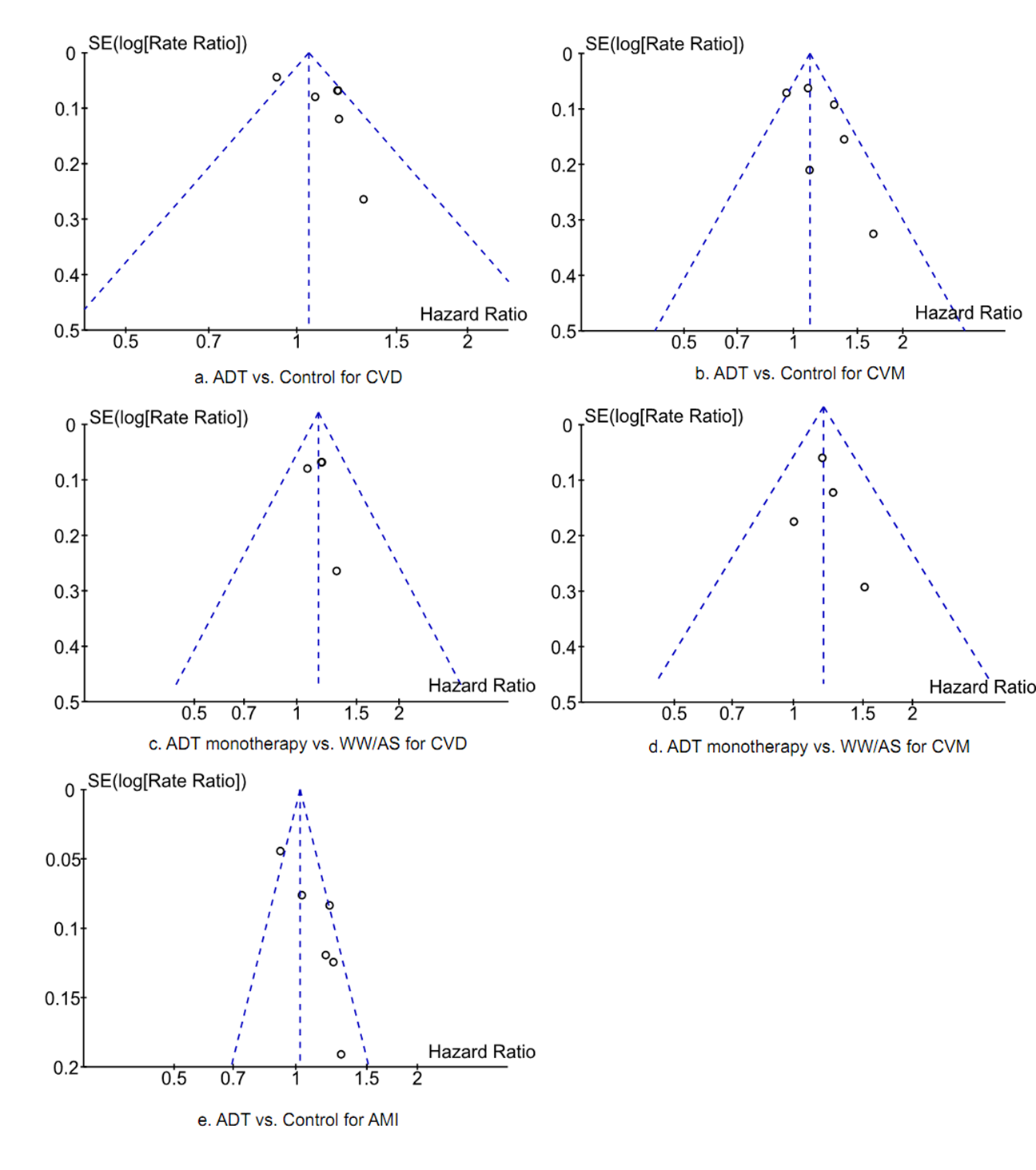


**Figure S7. Updated Meta-analysis from RCTs for CVM related to ADT**


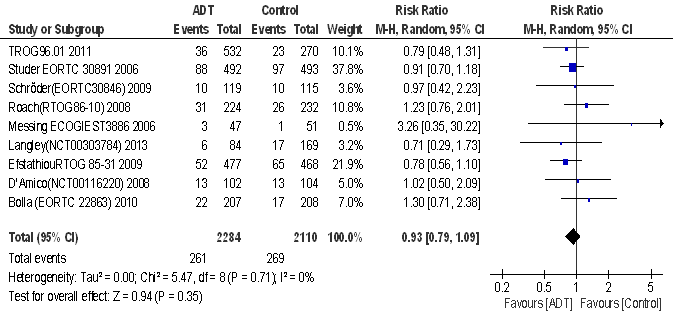

Supplement: File S1 — Containing the following contents. Methods S1. Literature Search Strategy. Table S1. List of Excluded Full-text Articles with Reasons for Exclusions, for Both CVD and CVM. Table S2. Newcastle-Ottawa Scale Quality Assessment of Included Studies, for both CVD and CVM. Table S3. Characteristics of Studies Investigating AMI Related to ADT. Table S4. Pooled Results and Publication Bias for All Comparisons. Figure S1. HRs of Subgroup Analyses for CVD and CVM Related to Different Types of ADT. Figure S2. Details of Subgroup Analyses for CVD Related to Different Types of ADT. Figure S3. Details of Subgroup Analyses for CVM Related to Different Types of ADT. Figure S4. HR of AMI Morbidity Related to ADT. Figure S5. Details of Subgroup Analyses for AMI Related to Different Types of ADT. Figure S6. Funnel Plots for All Meta-analyses. Figure S7. Updated Meta-analysis from RCTs for CVM related to ADT. (DOC) [file pone.0107516.s001.doc]
