# Supplementary material for: Androgen Deprivation Therapy for Prostate Cancer Is Associated with Cardiovascular Morbidity and Mortality: A Meta-Analysis of Population-Based Observational Studies
Source: PLoS One. 2014 Sep 29;9(9):e107516. doi: 10.1371/journal.pone.0107516 (PMC4180271; doi:10.1371/journal.pone.0107516)
Supplement: Checklist S1 — PRISMA checklist. Preferred Reporting Items for Systematic Reviews and Meta-Analyses. (DOC) [file pone.0107516.s002.doc]

| **Section/topic** | **#** | **Checklist item** | **Reported on page #** |
| --- | --- | --- | --- |
| **TITLE** | | |  |
| Title | 1 | Androgen Deprivation Therapy for Prostate Cancer is associated with Cardiovascular Morbidity and Mortality: A Meta-analysis of Population-based Observational Studies | Title |
| **ABSTRACT** | | |  |
| Structured summary | 2 | ***Background:*** There is no consensus regarding whether androgen deprivation therapy (ADT) is associated with cardiovascular disease (CVD) and cardiovascular mortality (CVM). The objective of this study was to determine the role of ADT for prostate cancer (PCa) in development of cardiovascular events (CVD and CVM).  ***Methods and Findings:*** We performed a meta-analysis from population-based observational studies comparing ADT vs control aimed at treating PCa in patients with PCa, reporting either CVD or CVM as outcome. Publications were searched using Medline, Embase, Cochrane Library Central Register of observational studies database up to May 31th 2014, and supplementary searches in publications from potentially relevant journals. 6 studies were identified with a total of 129,802 ADT users and 165,605 controls investigating the relationship between ADT and CVD. The incidence of CVD was 10% higher in ADT groups, although no signiﬁcant association was observed (HR=1.10, 95%CIs: 1.00-1.21; P=0.06). For different types of ADT, CVD was related with gonadotropin-releasing hormone (GnRH) (HR=1.19, 95%CIs: 1.04-1.36; P<0.001) and GnRH plus oral antiandrogen (AA) (HR=1.46, 95%CIs: 1.03-2.08; P=0.04), but not with AA alone or orchiectomy. For CVM, 119,625 ADT users and 150,974 controls from 6 eligible studies were included, pooled result suggested that ADT was associated with CVM (HR=1.17, 95%CIs: 1.04-1.32; P=0.01). Significantly increased CVM was also detected in GnRH and GnRH plus AA groups. When patients received other treatments (e.g. prostatectomy and radiotherapy) were ruled out of consideration, more increased CVD (HR=1.19, 95%CIs: 1.08-1.30; P＜0.001) and CVM (HR=1.30, 95%CIs: 1.13-1.50; P＜0.001) were found in men treated with ADT monotherapy.  ***Conclusions:*** ADT is associated with both CVD and CVM. Particularly, GnRH alone and GnRH plus AA can significantly increase the incidence of cardiovascular events in patients with PCa. | Abstract |
| **INTRODUCTION** | | |  |
| Rationale | 3 | If androgen deprivation therapy (ADT) increase the risk of cardiovascular events?  What kinds of ADT can increase CVD and CVM?  When patients received other treatments (e.g. prostatectomy and radiotherapy) were ruled out of consideration, if more steady increased CVD or CVM risk can be found in men treated with ADT monotherapy compared with watchful waiting or active surveillance (WW/AS)?  There is still no consensus regarding that ADT is associated with cardiovascular events. | Introduction |
| Objectives | 4 | A meta-analysis and system review was performed by our research group to test if ADT is associated with an increased risk of cardiovascular events in patients with PCa. | Introduction |
| **METHODS** | | |  |
| Protocol and registration | 5 | Indicate if a review protocol exists, if and where it can be accessed (e.g., Web address), and, if available, provide registration information including registration number. | Method： Search strategy and study selection |
| Eligibility criteria | 6 | 1) The type of studies should be population-based cohort or nested case-control without subjects-selection bias. So the case–control studies prone to recall and interviewer bias were excluded; 2) Patients diagnosed with PCa; 3) The intervention groups used ADT only or ADT plus other treatments; 4) The patients in control groups never received ADT; 5) Study must either report risk estimates with 95% Confidence intervals (CIs), or report sufficient data to estimate these; 6) Included studies had to provide comparative data. | Method：Search strategy and study selection |
| Information sources | 7 | References of included studies and narrative reviews were searched for potential studies. | Method：Search strategy |
| Search | 8 | Medline up to May 31^th^ 2014，EMBASE up to May 31^th^ 2014，and Cochrane Library database up to May 31^th^ 2014 | Search strategy |
| Study selection | 9 | Included studies were restricted to studies that should meet all of the following inclusion criteria: 1) Patients diagnosed with prostate cancer; 2) The intervention groups used ADT only or ADT plus other treatments; 3) The treatments in control groups were non-ADT (e.g. radical prostatectomy, radiotherapy, active surveillance, etc.); 4) Study must either report risk estimates with 95% CIs, or report sufficient data to estimate these; 5) Included studies had to provide comparative data. If more than one paper were identified from the same database, the most measurable (complete or recent) report of these articles was chosen for analysis. | Method：Study selection |
| Data collection process | 10 | All the data from eligible publications were carefully extracted independently by two authors (Zhao & Zhu), and all disagreements were resolved by the third reviewer (Niu) until consensus was achieved on all items. | Method：Data extraction and quality assessment |
| Data items | 11 | For each study included, the following information was considered: first author’s name, year and research institution of the study, study design, the number of case and control subjects, median age of patients, duration of follow-up, inclusion criteria, treatment in both groups, types and duration of ADT, definition of cardiovascular events, HRs or RRs and corresponding 95% CIs of estimates in each comparisons, or the informations required to calculate these. | Method：Data extraction and quality assessment |
| Risk of bias in individual studies | 12 | Publication bias was evaluated using Begg adjusted rank correlation test and Egger linear regression test. All statistical analyses were conducted using STATA (version 11.0; College Station, Texas) and Review Manage (version 5.2; The Cochrane Collaboration, Oxford). Two-tailed P<0.05 was considered statistically signiﬁcant. | Method：Statistical Analysis |
| Summary measures | 13 | The HRs were used to compare dichotomous variables, which was directly extracted if it was reported in the studies. Otherwise, we looked for the number of events in both ADT and control groups, the total number of patients in each group and the log-rank statistic or its P value allowing calculation of an approximation of the HR estimate. When two or more types of ADT from one study were respectively compared with the same control group (e.g. GnRH vs Control and AA vs Control), we used random effects meta-analysis to combine these results as necessary. | Method：Statistical Analysis |
| Synthesis of results | 14 | For the analysis of CVD related to ADT: HR=1.10; 95% CI, 1.00-1.21; *P*=0.06, *I*^2^=72%. For the analysis of CVM related to ADT: HR=1.17; 95% CI, 1.04-1.32; *P*=0.01, *I*^2^=57%. | Figure 2 |

Page 1 of 2

| **Section/topic** | **#** | **Checklist item** | **Reported on page #** |
| --- | --- | --- | --- |
| Risk of bias across studies | 15 | In our assessment of publication bias, funnel plots showed balance, with points distributing around the verticals, indicating no obvious publication bias. Additionally, actualized data from Begg’s and Egger’s tests also suppoted no exhibited publication bias. | Supplemental Content  Table S4  Figure S2 |
| Additional analyses | 16 | To minimize the influence of concomitant treatments (e.g. radiotherapy and prostatectomy), analyses of ADT monotherapy vs watchful waiting or active surveillance (WW/AS) for cardiovascular events were particularly performed. ADT monotherapy was defined as treatment in intervention group was only ADT without any other previous therapies for PCa. Considering the significance of existing heterogeneity in overall-analysis, additional subgroup-analyses for various types of ADT (e.g. GnRH, AA, GnRH+AA and Orchiectomy) vs non-ADT were performed to minimize the heterogeneity. | Method：Subgroups analyses |
| **RESULTS** | | |  |
| Study selection | 17 | Finally, seven studies were included in this meta-analysis (see Figure 1). All papers used in our analysis were observational studies and published in English. List of excluded full-text articles with reasons are shown in Table S1. | Literature search and characteristics of the included studies |
| Study characteristics | 18 | Details of the included studies were summarized in Table 1 and Table 2. | Table 1-2 |
| Risk of bias within studies | 19 | In our assessment of publication bias, funnel plots showed balance, with points distributing around the verticals, indicating no obvious publication bias (Figure S6). Additionally, actualized data from Begg’s and Egger’s tests also suppoted no exhibited publication bias (Table S4). | Results |
| Results of individual studies | 20 | For all outcomes considered (benefits or harms), present, for each study: (a) simple summary data for each intervention group (b) effect estimates and confidence intervals, ideally with a forest plot. | Table 1-2  Figure 2-3 |
| Synthesis of results | 21 | Among 129,802 ADT users, 23,126 (17.8%) developed CVD compared with 26,536 events (16.0%) among 165,605 non-ADT users (HR=1.10; 95% CI, 1.00-1.21; P=0.06; Figure 2a). A total of 13,995 CVM events happened in ADT group, accounting for 11.7% of ADT users. In control group, there were 13,645 CVM events, with the overall incidence of 9.0%. Overall analysis showed that ADT was significantly associated with CVM (HR=1.17; 95% CI, 1.04-1.32; P=0.01; Figure 2b). | Results |
| Risk of bias across studies | 22 | As in almost all of the meta-analyses, results may be influenced by selection bias. In our study, predesigned search strategy was performed to minimize selection bias as extensive as we could, with independent selection and data extraction by two separate reviewers. | Discussion |
| Additional analysis | 23 | In order to minimize the influence of prior treatments, we attempted to exclude patients received radiotherapy or prostatectomy or studies with results not already adjusted for the primary therapy. Then comparison of ADT monotherapy vs WW/AS was addressed. | Results |
| **DISCUSSION** | | |  |
| Summary of evidence | 24 | The results of quality assessment according to NOS for cohort and case-control studies were shown in Table S2. All eligible studies were of high quality based on NOS with scores ranged from seven to nine stars. LOE of included studies were all 2a. | Supplemental Content |
| Limitations | 25 | Firstly, as in almost all of the meta-analyses, results may be influenced by selection bias. In our study, predesigned search strategy was performed to minimize selection bias as extensive as we could, with independent selection and data extraction by 2 separate reviewers. Studies were included without any language restriction and including registries. Secondly, incomplete data in some included studies may influence the overall result of our study to some extent. HRs or RRs were not directly given in some papers, we had to calculate them through the number of cardiovascular events in both ADT and control groups as a result; when two or more types of ADT groups from one study were respectively compared with the same control group (e.g. AA alone vs control, GnRH alone vs control), we used random effect meta-analysis to combine these different data together for compositing overall result. Thirdly, the diversity of cardiovascular events definitions in different studies may affect the results of overall analyses. In order to reduce the bias, confirmatory subgroup analysis for AMI risk was conducted. Furthermore, the certain characteristics of patients (e.g. age, pathologic stages, comorbidities, ADT duration etc.) that may contribute to cardiovascular events were different in each included study, which might substantially confound the presented results. So, adjusted data were extracted when available to minimize the bias. | Discussion |
| Conclusions | 26 | In conclusion, pooled result shows that ADT could significantly increase the risk of CVM. Although no significant association is observed between ADT and CVD, there is still a tendency favoring non-ADT users. After removing patients received other treatments, such as prostatectomy and radiotherapy, much stronger associations of ADT with CVD and CVM are observed. Moreover, subgroup analyses for different types of ADT suggest that GnRH and GnRH plus AA, but not AA alone or orchidectomy, can significantly lead to both CVD and CVM. These findings may help clinicians make clinical decision when prescribing ADT. Additional studies are needed to further define populations for whom benefits from ADT outweigh risks and to develop strategies to prevent ADT-related cardiovascular events. | Discussion |
| **FUNDING** | | |  |
| Funding | 27 | The work was supported by the National Basic Research Program of China (grant no. 2012CB518304) and the International S&T Cooperation Program of China (ISTCP) (grant no. S2012GR0142). Role of the Sponsor: The sponsor had no role in the design and conduct of the study; the collection, management, analysis, and interpretation of the data; the preparation, review, or approval of the manuscript; or the decision to submit the manuscript for publication.' | Funding |

*From:*  Moher D, Liberati A, Tetzlaff J, Altman DG, The PRISMA Group (2009). Preferred Reporting Items for Systematic Reviews and Meta-Analyses: The PRISMA Statement. PLoS Med 6(6): e1000097. doi:10.1371/journal.pmed1000097

For more information, visit: **www.prisma-statement.org**.

Page 2 of 2
