# Supplementary material for: Androgen Deprivation Therapy for Prostate Cancer Is Associated with Cardiovascular Morbidity and Mortality: A Meta-Analysis of Population-Based Observational Studies
Source: PLoS One. 2014 Sep 29;9(9):e107516. doi: 10.1371/journal.pone.0107516 (PMC4180271; doi:10.1371/journal.pone.0107516)
Supplement: Checklist S2 — MOOSE checklist. Meta-analysis Of Observational Studies in Epidemiology. (DOC) [file pone.0107516.s003.doc]

**MOOSE Checklist**

From: [Donna F. Stroup](http://jama.ama-assn.org/search?author1=Donna+F.+Stroup&sortspec=date&submit=Submit), PhD, MSc; [Jesse A. Berlin](http://jama.ama-assn.org/search?author1=Jesse+A.+Berlin&sortspec=date&submit=Submit), ScD; [Sally C. Morton](http://jama.ama-assn.org/search?author1=Sally+C.+Morton&sortspec=date&submit=Submit), PhD; [Ingram Olkin](http://jama.ama-assn.org/search?author1=Ingram+Olkin&sortspec=date&submit=Submit), PhD; [G. David Williamson](http://jama.ama-assn.org/search?author1=G.+David+Williamson&sortspec=date&submit=Submit), PhD; [Drummond Rennie](http://jama.ama-assn.org/search?author1=Drummond+Rennie&sortspec=date&submit=Submit), MD; [David Moher](http://jama.ama-assn.org/search?author1=David+Moher&sortspec=date&submit=Submit), MSc; [Betsy J. Becker](http://jama.ama-assn.org/search?author1=Betsy+J.+Becker&sortspec=date&submit=Submit), PhD; [Theresa Ann Sipe](http://jama.ama-assn.org/search?author1=Theresa+Ann+Sipe&sortspec=date&submit=Submit), PhD; [Stephen B. Thacker](http://jama.ama-assn.org/search?author1=Stephen+B.+Thacker&sortspec=date&submit=Submit), MD, MSc; for the Meta-analysis Of Observational Studies in Epidemiology (MOOSE) Group. **Meta-analysis of Observational Studies in Epidemiology. A Proposal for Reporting** JAMA. 2000;283(15):2008-2012. doi: 10.1001/jama.283.15.2008

|  | Reported on page | | Comments |
| --- | --- | --- | --- |
| **Reporting of background should include** | | | |
| Problem definition | Introduction | | If androgen deprivation therapy (ADT) increase the risk of cardiovascular disease (CVD) or cardiovascular mortality (CVM)?  What kinds of ADT can increase CVD or CVM incidence?  When patients received other treatments (e.g. prostatectomy and radiotherapy) were ruled out of consideration, if more steady increased CVD or CVM risk can be found in men treated with ADT monotherapy compared with watchful waiting or active surveillance (WW/AS)?  There is still no consensus regarding that ADT is associated with CVD and CVM. Although the latest meta-analysis of RCTs [drew](app:ds:draw) [a](app:ds:a) non-significant [conclusion](app:ds:conclusion) that ADT tended to be associated with an increased risk of CVM. But the validity of the results was suspected because of many biases. For purpose of investigating the rare adverse reaction, the credibility is much higher to perform a meta-analysis of large-scale observational studies. |
| Hypothesis statement | Introduction | | ADT maybe associated with higher risk of CVD and CVM than controls. |
| Description of study outcomes | Methods | | Hazard ratios (HRs) or risk ratios (RRs) of CVD and CVM among patients undergoing ADT compared to controls. |
| Type of exposure or intervention used | Methods | | The following types of ADT:  Gonadotropin-releasing hormone (GnRH) agonists  Oral antiandrogens (AA)  Orchiectomy  Combined ADT (two or more types above combined) |
| Type of study designs used | Methods: Search Strategy and Study Selection | | Population based cohort studies and nested case-control studies without subjects-selection bias, examining the prevalence of cardiovascular events (CVD or CVM) in ADT users and controls. |
| Study population | Methods: Search Strategy and Study Selection | | Patients included in individual studies were diagnosed with diagnosed with prostate cancer (PCa).  The patients in control groups never received ADT. |
| **Reporting of search strategy should include** | | | |
| Qualifications of searchers (e.g. librarians and investigators) | | Methods: Data Extraction and Quality Assessment | Dr Zhao was trained in systematic methods of literature searching as part of his PhD studies, Tianjin Medical university. Search methodology was discussed with Zhu, Department of Urology, Second Hospital of Tianjin Medical Unversity, Tianjin Institute of Urology, he has 2 years-experience of assisting research/academic staff develop search strategies for systematic reviews |
| Search strategy, including time period used in the synthesis and key words | | Methods: Search Strategy | Medline from 1966 – December 2013  EMBASE from 1974 – December 2013  Cochrane Library database through December 2013  See methodology and emethods |
| Effort to include all available studies, including contact with authors | | Methods: Search Strategy | There was no language, publication year, or other limit used.  Authors were not contacted, as adequate information for the performance of this review was available from studies and abstracts |
| Databases and registries searched | | Methods: Search Strategy | Medline, EMBASE and Cochrane Library, see emethods |
| Search software used, name and version, including special features used (e.g. explosion) | | Methods: Search Strategy | PubMed was accessed from the National Library of Medicine (free online)  Medline and Embase are available on the OVID SP platform  Cochrane Library is available on the Wiley Interscience platform. |
| Use of hand searching (e.g. reference lists of obtained articles) | | Methods: Search Strategy | References of included studies and narrative reviews were searched for potential studies. |
| List of citations located and those excluded, including justification | | Methods: Quality Assessment | Details of the literature search process are outlined in the PRISMA flow chart (Figure 1).  List of excluded full-text articles with reasons are shown in Table S1. |
| Method of addressing articles published in languages other than English | |  | We placed no restrictions on language; We were able to obtained all articles potentially eligible for inclusion in English language |
| Method of handling abstracts and unpublished studies | |  | No relevant unpublished data or abstract were observed. |
| Description of any contact with authors | |  | Contact was not made with authors of the studies because data was adequate to calculate HR or RR that was performed for our meta-analysis. We analyzed incidences by types of ADT, where the data wasn’t provided by some studies, but we did not pursue it in all studies as this was a secondary aim of this review. |
| **Reporting of methods should include** | | | |
| Description of relevance or appropriateness of studies assembled for assessing the hypothesis to be tested | |  | Detailed inclusion and exclusion criteria are described in the paper.  Study methodology is well documented.  If more than one paper were identified from the same database, the most measurable (complete or recent) report of these articles was chosen for analysis.  All papers used in our analysis were published in English. |
| Rationale for the selection and coding of data (e.g. sound clinical principles or convenience) | |  | Studies were included and excluded as per criteria outlined above.  Zhao and Zhu extracted data from each study according to a piloted proforma (see the method of Data Extraction in Manuscript text). Niu supervised and validated the piloting process. |
| Documentation of how data were classified and coded (e.g. multiple raters, blinding and inter-rater reliability) | |  | After data extraction and calculation of individual study estimates (HRs and 95% CIs as outlined above) by Zhao. All data extraction validated by Zhu (blindly) with full agreement.  HRs and 95% CIs of cardiovascular events were calculated according to different methods (see the method of Data Extraction in Manuscript text) from crude data extracted; initially by Zhao and then exact rates were calculated through the STATA statistical package by Zhu.  Year was documented as final-year of study |
| Assessment of confounding (e.g. comparability of cases and controls in studies where appropriate) | | Methods: Statistical Analysis | The adjusted HRs and 95% CIs reported in the studies were directly extracted. If the adjusted HRs were not directly given in the study, all the data both in cases and controls, available to calculate HRs, were adjusted for the different durations of follow-up.  We conducted subgroup analyses of different types of ADT vs control. |
| Assessment of study quality, including blinding of quality assessors, stratification or regression on possible predictors of study results | | Methods: Quality Assessment | We used a modified version of the Newcastle Ottawa Scale (NOS) to assess the quality of each study. The observational studies were considered to be of high-quality if it achieved more than six stars. |
| Assessment of heterogeneity | |  | We used Q and I^2^ statistics to assess heterogeneity |
| Description of statistical methods (e.g. complete description of fixed or random effects models, justification of whether the chosen models account for predictors of study results, dose-response models, or cumulative meta-analysis) in sufficient detail to be replicated | | Methods: Quality Assessment and Statistical Analysis | The HRs were used to compare all dichotomous variables. We used different methods to estimate the HRs according to the data provided in the publications. The HRs and 95% CIs reported in the studies were directly extracted. If those data were not available, we looked for the number of events in both ADT and control groups, the total number of patients in each group and the log-rank statistic or its P value allowing calculation of an approximation of the HR estimate. If data were only available in the form of survival curve, we extracted from the survival rates at some speciﬁed times so as to estimate the HRs value and its variance, assuming that the rate of patients censored was constant during the study follow-up. If the data mentioned above were not available, we had to consider estimating the RRs at last. When two or more types of ADT from one study were respectively compared with the same control group (e.g. GnRH vs Control and AA vs Control), we used random effects meta-analysis to combine these different types of ADT groups as necessary.  Pooled estimates were calculated using random effects models from Review Manager 5.2  Cochrane’s Q statistic was used to assess the statistical heterogeneity between included studies. Additionally, inconsistency was quantiﬁed by I^2^ statistic (100%×[(Q-df)/Q]), higher value denoting greater degree of heterogeneity.  With the Der-Simonian and Laird method, random-effects model was used no matter whether heterogeneity was observed or not.  Publication bias was evaluated using Begg adjusted rank correlation test and Egger linear regression test with STATA (version 11.0; College Station, Texas) |
| Provision of appropriate tables and graphics | |  | We included PRISMA flow chart to show the method of studies identification, Table1,Table2 and Table S3 showing characteristics of included studies, Table S2 showing results of quality assessment, Table S4 showing the pooled results and publication bias for all comparisons. Four forest plots of the main different meta-analyses conducted and five Funnel plots for publication bias assessment. |
| **Reporting of results should include** | | | |
| Graphic summarizing individual study estimates and overall estimate | |  | Figure 2, Figure 3, Figure S1, Figure S4, and Figure S5 |
| Table giving descriptive information for each study included | |  | Table 1, Table 2, and Table S3 |
| Results of sensitivity testing (e.g. subgroup analysis) | |  | We conducted analysis of ADT monotherapy vs watchful waiting or active surveillance (WW/AS) for cardiovascular events. (Figure 3) |
| Indication of statistical uncertainty of findings | |  | 95% confidence intervals intervals were presented for all analyses together with I^2^ values for the main meta-analyses |
| **Reporting of discussion should include** | | | |
| Quantitative assessment of bias (e.g. publication bias) | | Results: Publication Bias | Results of subgroup analyses are discussed with main potential confounding factors discussed. funnel plots showed balance, with points distributing around the verticals, indicating no obvious publication bias (Figure S6). Additionally, actualized data from Begg’s and Egger’s tests also suppoted no exhibited publication bias (Table S4). |
| Justification for exclusion (e.g. exclusion of non-English language citations) | |  | Reasons for exclusion were reported mainly in the results section, with the two main reasons being either that studies were irrelevant (Did not measure outcome of interest) or did not have clear information with no extra data obtainable. List of excluded full-text articles with reasons are shown in Table S1. |
| Assessment of quality of included studies | | Results: Characteristics of the Included Studies | The results of quality assessment according to NOS were shown in Table S2. All eligible studies were of high quality based on NOS with scores ranged from six to nine stars. |
| **Reporting of conclusions should include** | | | |
| Consideration of alternative explanations for observed results | | Discussion | See discussion. |
| Generalization of the conclusions (e.g. appropriate for the data presented and within the domain of the literature review) | | Discussion | Pooled result shows that ADT could significantly increase the risk of CVM. Although no significant association is observed between ADT and CVD, there is still a tendency favoring non-ADT users. After removing patients received other treatments, such as prostatectomy and radiotherapy, much stronger associations of ADT with CVD and CVM are observed. Moreover, subgroup analyses for different types of ADT suggest that GnRH and GnRH plus AA, but not AA alone or orchidectomy, can significantly lead to both CVD and CVM. |
| Guidelines for future research | | Conclusion | These findings may help clinicians make clinical decision when prescribing ADT. Additional studies are needed to further define populations for whom benefits from ADT outweigh risks and to develop strategies to prevent ADT-related cardiovascular events. |
| Disclosure of funding source | |  | The work was supported by the National Basic Research Program of China (grant no. 2012CB518304) and the International S&T Cooperation Program of China (ISTCP) (grant no. S2012GR0142). The sponsor had no role in the design and conduct of the study; the collection, management, analysis, and interpretation of the data; the preparation, review, or approval of the manuscript; or the decision to submit the manuscript for publication. |

Transcribed from the original paper within the Support Unit for Research Evidence (SURE), Cardiff University, United Kingdom. February 2011.
